# Transcriptome analysis reveals the expression of genes involved in the formation of petal variegation in *Impatiens morsei*

**DOI:** 10.1038/s41598-025-93846-0

**Published:** 2025-04-02

**Authors:** Hai-Ge Liu, Ting-Ting Feng, Si-Yu Ren, Jian-Yuan Yang, Lu-Qiu Zhao, Xiao-Li Zhang, Mei-Juan Huang, Hai-Quan Huang

**Affiliations:** 1 College of Landscape Architecture and Horticulture Sciences, Southwest Forestry University, Yunnan, 650224 China; 2 Southwest Research Center for Engineering Technology of Landscape Architecture(State Forestry and Grassland Administration), Yunnan, 650224 China; 3 Yunnan Engineering Research Center for Functional Flower Resources and Industrialization, Yunnan, 650224 China; 4 Research and Development Center of Landscape Plants and Horticulture Flowers, Yunnan, 650224 China

**Keywords:** *Impatiens morsei*, Variegation, Transcriptome analysis, Gene expression, Molecular biology, Transcriptomics

## Abstract

*Impatiens morsei* is an annual or perennial flower that is a member of the Impatiens genus in the Balsaminaceae family, with pink flowers and dark purple spots on the vexil and wing petals, which is important for the study of new flower colors of Balsaminaceae. However, there has been no study on the flavonoid biosynthesis pathway and carotenoid biosynthesis pathway of *I. morsei* and the mechanism of its pigment formation is still unclear. Transcriptome sequencing of spotted and non-spotted regions was performed using the petals of *I. morsei* at full bloom as test material. The results showed that (1) After transcriptome sequencing, 39,917 unigene sequences and 39,110 differentially expressed genes were obtained, and the number of genes that are down-expressed was higher than genes that are up-expressed. (2) Three key genes in the flavonoid biosynthetic pathway, *FLS*, *PAL*, and *CHS1*, were screened out. Additionally, two key genes in the carotenoid biosynthetic pathway, *ABA1* and *CCS*, were identified. Furthermore, two regulatory genes, MSI4 and CPC, were also selected.and analyzed the spatiotemporal expression patterns of these seven genes. The results of this study lay a theoretical foundation for the study of the formation mechanism of floral spots in *I. morsei*.

**Supplementary Information:**

The online version contains supplementary material available at 10.1038/s41598-025-93846-0.

## Introduction

Flower color is a crucial characteristic of ornamental plants, and it consistently remains a focal point in biological studies^1^. In addition to the common monochromatic and bicolored flowers, there is a special trait in flower coloration known as petal variegation, which is widely present in flowering plants and plays an important ecological role in plant pollination, defense, and adaptation to environmental changes. Flower spots are not only an important ornamental trait for garden plants but also an ideal entry point for studying the fine regulation and differential Gene expression associated with flower color^2,3^.The phenomenon of plant variegation was first recorded by the Roman naturalist Pliny the Elder (23–79 AD), who documented the existence of variegated ivy. However, no in-depth studies were conducted on variegation until the mid-19th century, when the discovery of chloroplasts and research into their origins began to draw attention to this phenomenon^4^.Plant variegation can be categorized into two main types: regular and irregular^5^. Variegation plays important ecological roles in attracting pollinators and deterring herbivores. For example, the intricate petal spots of the beetle daisy (Gorteria diffusa) mimic female hoverflies to attract male flies through sexual deception, thereby enhancing pollination efficiency^6^. Extensive research indicates that that variegation is primarily caused by uneven pigment distribution, variegated areas contain anthocyanins, while non-variegated areas lack these pigments^7–9^. Research has demonstrated that carotenoids, betalains, and anthocyanins are the primary pigments responsible for determining flower color in plants^10^. Anthocyanins, a subclass of flavonoids, are water-soluble flavonoid compounds and have been shown to play a crucial role in flower color formation^11^. The most important anthocyanins identified include pelargonidin, cyanidin, delphinidin, peonidin, petunidin, and malvidin. These anthocyanins are responsible for the red, orange, blue, and purple colors of flowers^12^. Carotenoids are present in the plastids of floral cells, exhibiting colors varying from yellow to orange^13^. Betalain appear yellow to red^14^. Flower color phenotype traits are influenced due to numerous factors, including the type and content of pigments in the petals, the pH of the cell sap, the shape of the petal cell epidermis, the internal and external physical structures of the petals, and the genes controlling flower color expression development^15^.

Genetic factors are instrumental in the production of anthocyanins, with the regulation of these genes being tightly correlated with anthocyanin levels, which subsequently impacts the coloration of plant flowers^16^ The production of anthocyanins, which is a part of the flavonoid synthesis pathway, is collectively controlled by structural genes, regulatory genes, and environmental influences. Chalcone synthase (CHS) as the first key enzyme catalyzing the biosynthesis pathway of anthocyanins, was identified in *Petroselinum hortense*^17^. Chalcone synthase genes have been cloned from a variety of plants, such as *Arabidopsis thaliana*, *Paeonia lactiflora*, *Petunia hybrida*, *Glycine max*, and *Solanum lycopersicum*, among others^18^. Studies showed that the post-transcriptional inhibition of the *CHS* gene participated in the formation of white petals in *Dahlia pinnata*^19^. In the study related to *Impatiens uliginosa*, Zhao et al. cloned three copies of the *CHS* gene. The expression of these three *CHS* genes only showed significant differences in the pink flowers, with the highest expression level observed in the *CHS1* gene^20^. Reports have indicated the presence of phenylalanine ammonia-lyase (PAL) in the majority of plants and microorganisms, first discovered by Koukol et al. in *Hordeum vulgare* L^21^. Subsequently, extensive research was conducted, and among the plants for which related genes have been reported to be cloned are *Arabidopsis thaliana*, *Solanum tuberosum* L, *Brassica napus* L, *Lilium lancifolium* Thunb, and *Citrus reticulata* Blanco^22–26^. Studies had demonstrated that the activity of the *PAL* gene determined the synthesis of pigments such as anthocyanidins in the colored flowers of plants^27^. The precursor for anthocyanin production is phenylalanine, or 2-amino phenylalanine, which gets transformed into cinnamic acid (cinnamate) through the catalytic activity of phenylalanine ammonia-lyase (PAL), with PAL being the key enzyme that initiates anthocyanin biosynthesis^28^. Additionally, the flavonol synthase gene (*FLS*) was reported to have an impact on the accumulation of anthocyanins, the synthesis of flavonols, and coloration in plants^29^. FLS belongs to the 2-oxoglutarate-dependent dioxygenase (2-ODD) family. Tanaka and colleagues first isolated and cloned the *FLS* gene in *Petunia hybrida* and also successfully expressed it in yeast^30^. Wu et al. in their investigation into the influence of *FLS* on the flower color formation in the *genus Magnolia*, found that the expression of *FLS* genes exhibited clear tissue specificity, with *YdFLS* having the highest expression in flowers and *YlFLS* having the highest expression in young leaves^31^. The *FLS* gene was functionally conserved in the process of flower color formation in the *genus Magnolia*, which it played a role in promoting the synthesis of flavonols, and the differences in its transcription levels were the main reason for the formation of various flower colors. Scholars have also explored that the substrates for the *ANS* gene and the *FLS* gene are consistent, indicating that there is a competitive relationship and that the amino acid sequences of the two genes are also highly similar^32^. This has further confirmed that the *FLS* gene has an impact on the combination of both flavonols and anthocyanins in plants.

Anthocyanin synthesis governed not just by structural genes but also by structural genes but also by regulatory factors from the MYB, bHLH, and WD40 families^33,34^. The mechanism involves transcription factors controlling structural genes to further regulate plant flower color. Researchers found that R3-MYB class negative regulatory factors of the CPC-like type interacted with R2R3-MYB positive regulatory factors and bHLH proteins in a competitive manner, inhibiting the formation of the MBW complex, and further inhibiting anthocyanin biosynthesis^35^. For example, in *Arabidopsis thaliana*, the R3-MYB and *AtCPC* acted as transcriptional repressors of anthocyanin synthesis, competing with *AtPAP2* to negatively regulate the synthesis of anthocyanins. Researcher Zhu has experimentally confirmed that CPC acts as an inhibitory regulator in the biosynthesis of anthocyanins. CPC contends with the R2R3-MYB transcription factors PAP1/2, known as activators of genes involved in anthocyanin production^36^.

Over the past few years, there has been a growing amount of reports on the biosynthesis of carotenoids, and the expression of structural genes in their biosynthetic way has a certain impact on the structure and concentration of carotenoids in plants. Abscisic Acid (ABA) is an important plant hormone derived from carotenoids, and Zeaxanthin Epoxidase (ZEP) plays a significant role in the biosynthesis process of ABA^37^. The primary function of ZEP is to catalyze the conversion of zeaxanthin into antheraxanthin, which is further epoxidized to form violaxanthin^38^. The gene *ABA1* belongs to the ZEP family and is involved in these processes. In *Arabidopsis thaliana*, the biosynthesis of *ABA* begins with the enzyme ABA DEFICIENT 1 (ABA1) catalyzing the epoxidation of antheraxanthin, leading to the production of epoxycarotenoids, such as violaxanthin^39^. Deruere et al. first cloned the capsanthin-capsorubin synthase gene (*CCS*) from chili peppers and demonstrated that it was a single copy in the genome^40^. Milena and colleagues transferred the capsanthin-capsorubin synthase gene (*CCS*) from the decorative plant *Lilium lancifolium* into another decorative plant, *V. cornuta*^41^. The successful incorporation of the foreign gene enabled the production and buildup of red pigments within the petals, leading to a change in their coloration.

**Fig. 1:**
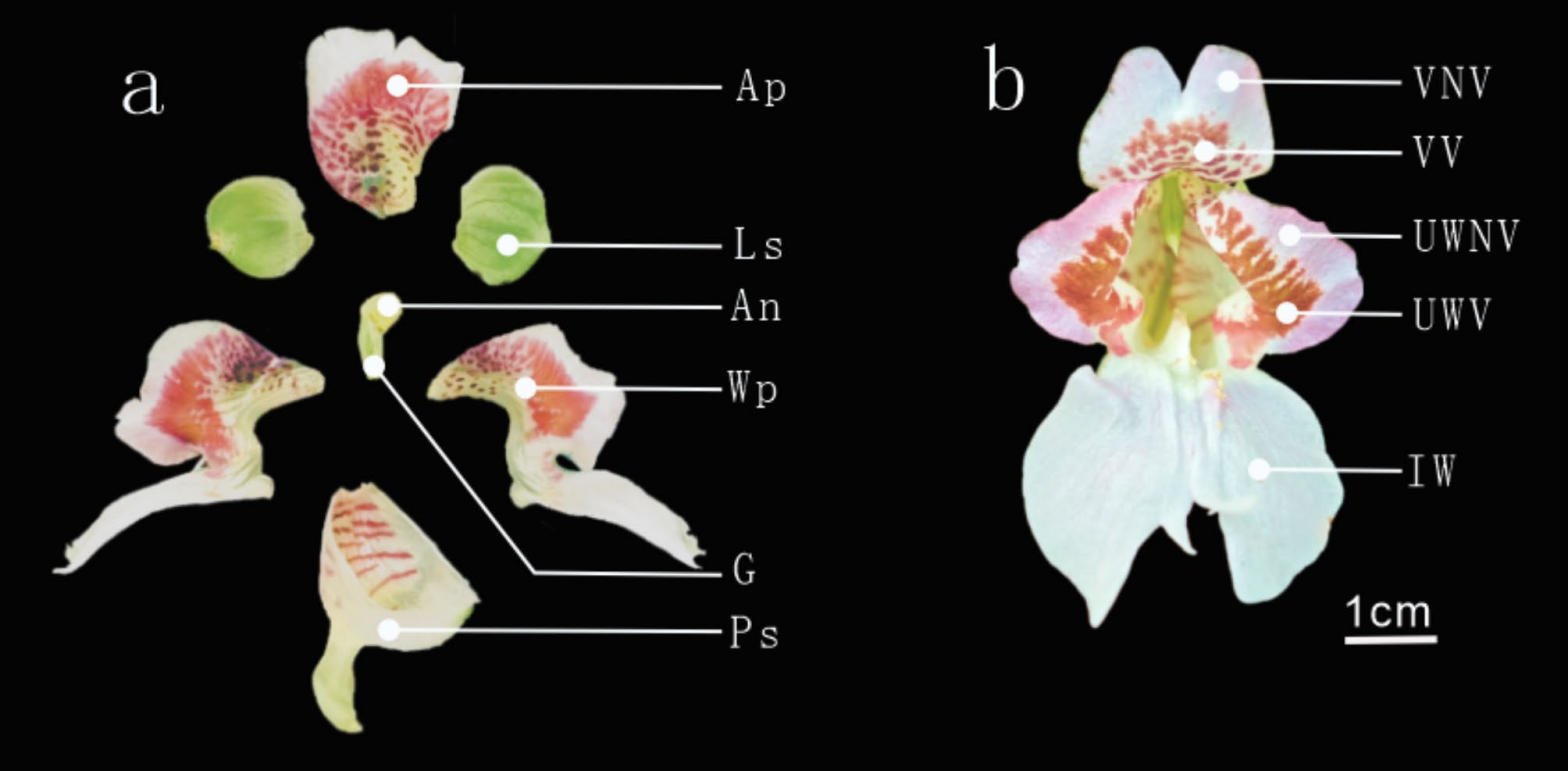
The flower basic structure diagram of *I. morsei*. (**a**): Ap: Anterior petal, Ls: Lateral sepal, An: Androecium, Wp: wing petal, G: Gynoecium, Ps: Posterior sepal. (**b**): VV: vexilla variegation; VNV: vexilla non-variegation; UWV: upper wing variegation; UWNV: upper wing non-variegation; IW: inferolateral wing;.

The experimental material of this study was *I. morsei* (Fig. 1), which is an annual or perennia herbaceous plant of the genus *Impatiens* in the family Balsaminaceae, mainly distributed in Guangxi. *I morsei* is characterized by its large and brightly colored flowers with orange-red spots and patches on the petals, which greatly enhance its ornamental value. However, the mechanism underlying the formation of these colorful spots in *I morsei* has not yet been reported. Therefore, identifying the genes related to spot formation and elucidating the mechanisms of spot development in *I morsei* will help deepen the understanding of the complexity and accuracy of plant gene expression. This research is also of great significance for breeding *Impatiens* varieties with unique spot patterns and for enhancing the ornamental and market value of *I morsei*.

## Results

### Unigene functional annotation and classification

All transcripts obtained from this transcriptome were compared with the six major databases: NR, Swiss-Prot, Pfam, COG, GO, and KEGG, to retrieve information from these databases. A total of 39,514 mRNA annotations were obtained, along with 24,420 Unigene annotation results, which accounted for 61. 80% of the total number of Unigene (Table S1).

The *I. morsei* transcriptome includes 21,088 GO-annotated unigenes (53.37% of total)(Figure S1). Among 16 biological processes, binding (11,118) and catalytic activity (10,355) are most abundant. In cellular components (13 items), cell part (11,212), membrane component (7,259), and organelle (6,543) are most represented. In molecular functions (23 items), cellular processes (8,890) and metabolic processes (7,375) have the most unigenes.In KEGG annotation(Figure S2), 10,759 Unigenes were categorized (27.23% of total) into six branches (Metabolism, Genetic Information Processing, Environmental Information Processing, Cellular Processes, Organismal Systems, and Human Diseases), Within the Metabolism category, the most Unigenes were involved in Carbohydrate Metabolism, with a total of 872; within Genetic Information Processing, the most unigenes were involved in Transcription, with 899. For COG annotation(Figure S3), 22,178 Unigenes were annotated (56.13% of total), with the highest number in the S-category (Function unknown, 11,771). The NR database comparison revealed(Figure S4) that *I. morsei* has high sequence similarity with species like *Camellia sinensis*, aiding in understanding its homologous sequences. In total, 24,209 Unigenes (61.27% of the annotation) were NR-annotated and distributed across 445 species. The top three species with the most annotated genes were *Camellia sinensis* (4,686, 19.29%), *Actinidia chinensis* (2,664, 10.97%), and *Nyssa sinensis* (2,476, 10.19%).

### Inter-sample expression analysis

**Fig. 2:**
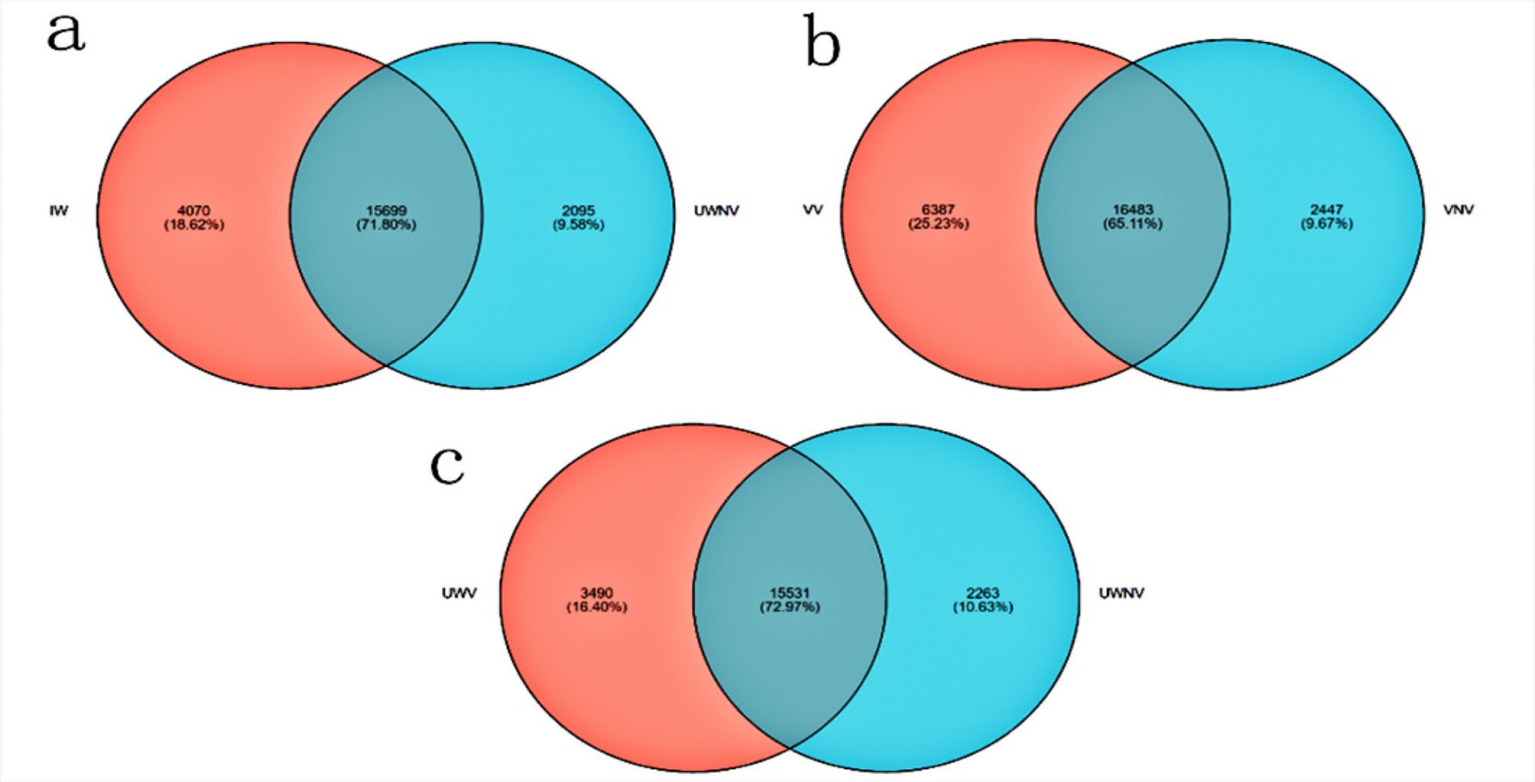
Venn analysis between samples (**a**): IW vs. UWNV. (**b**): VV vs. VNV.(**c**): UWV vs. UWNV.

Based on the different colors of the vexil and wing petal in the petals of *I. morsei*, they were divided into five distinct coloring regions, named VV, VNV, UWV, UWNV, and IW, the results showed there was observed that in the samples of VV and VNV, there were 6,387 unique genes in VV, 2,447 unique genes in VNV, and 16,483 genes common to both VV and VNV. In the samples of UWV and UWNV, there were 3,490 unique genes in UWV, 2,263 unique genes in UWNV, and 15,531 genes common to both UWV and UWNV. Between the IW and UWNV samples, there were 4,070 unique genes in IW, 2,095 unique genes in UWNV, and 15,699 genes common to both IW and UWNV(Fig. 2).

### Differential Gene(DEGs) expression analysis

**Fig. 3:**
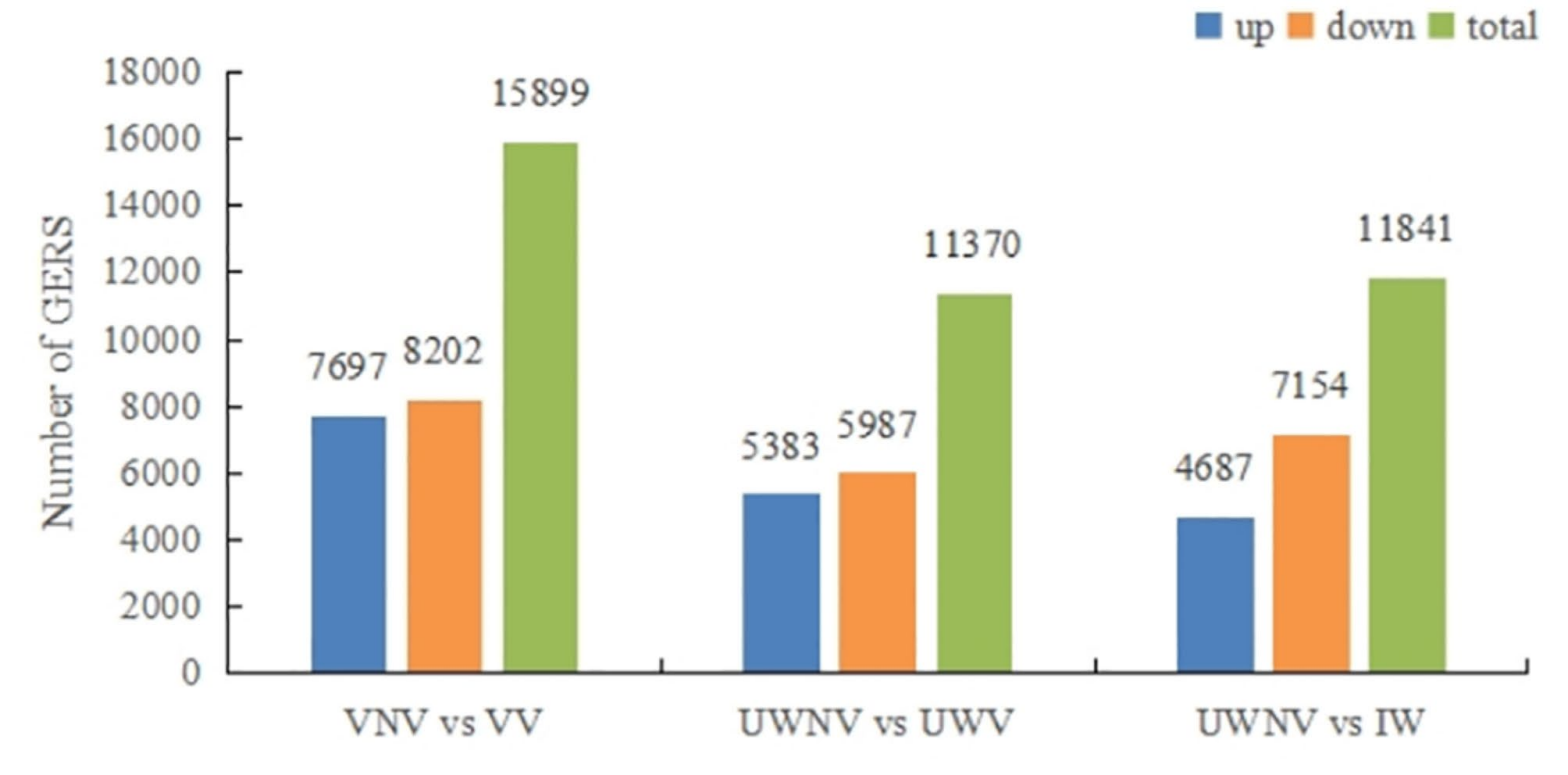
Up regulation and down regulation of differentially. expressed genes in different comparisons.

**Fig. 4:**
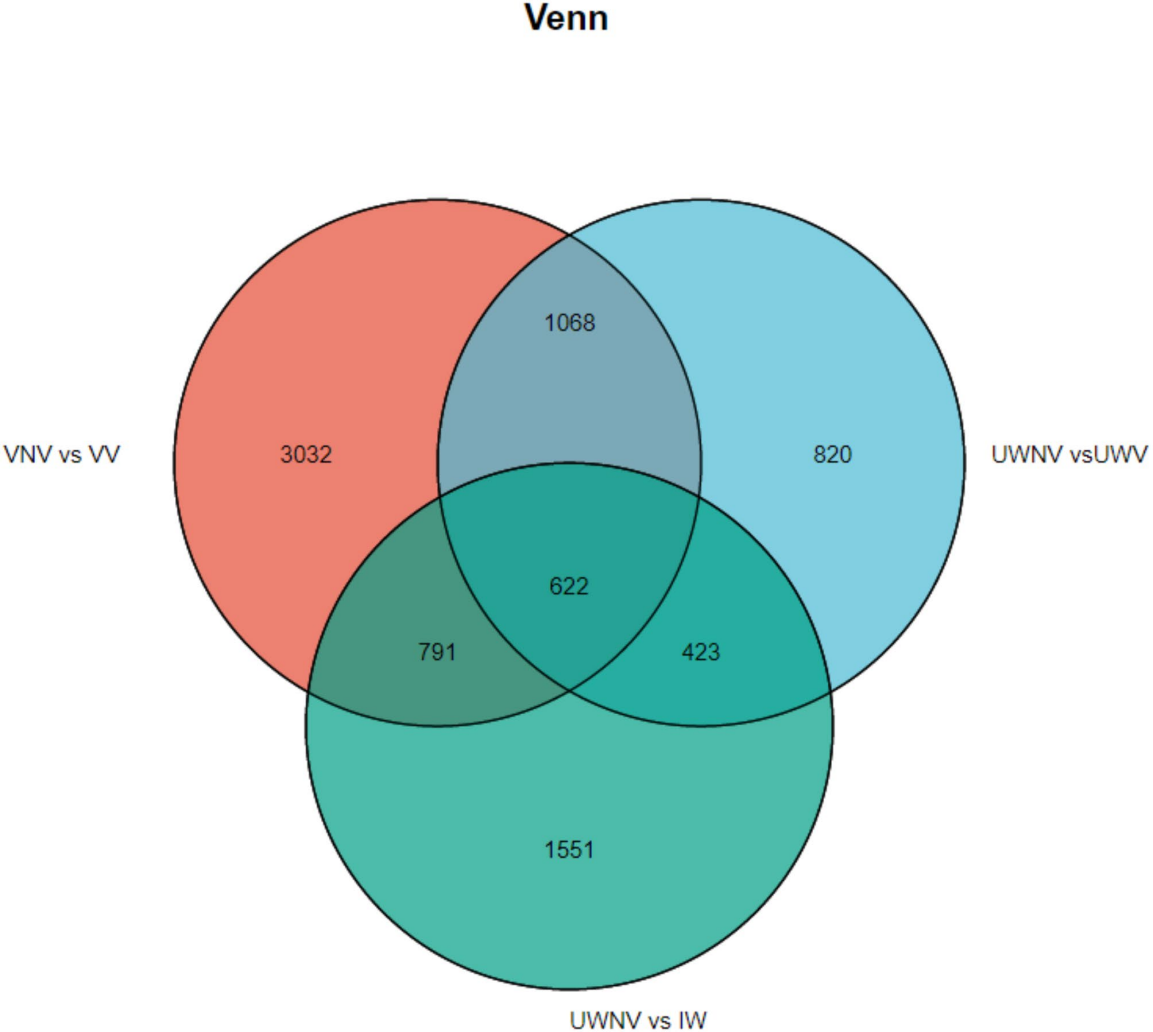
Venn diagram of differently expressed genes between different control groups.

Differential gene expression analysis was conducted using the criteria of |log2| > 1 and a significance level of < 0. 005 to filter for differentially expressed genes. Based on the different colors of the vexilla and wing petals in *I. morsei*, pairwise comparisons were made, which were divided into three groups: VNV vs. VV, UWNV vs. UWV and UWNV vs. IW. There were observed that a total of 17,767 genes were upregulated, and 21,343 genes were downregulated. The most differentially expressed genes were found in the comparison between VNV and VV, with 15,899 genes; followed by the comparison between UWNV and IW, with 11,841 genes; and the least number of differentially expressed genes were found in the comparison between UWNV and UWV, with 11,370 genes. In the comparisons of VNV vs. VV, UWNV vs. UW, and UWNV vs. IW, the majority of genes showed downregulation. Overall, there were more downregulated genes than upregulated ones (Fig. 3). It was observed that among all the differential genes, 622 Unigenes were common differential genes across all groups (Fig. 4).

### Differential gene expression GO annotation analysis

**Fig. 5:**
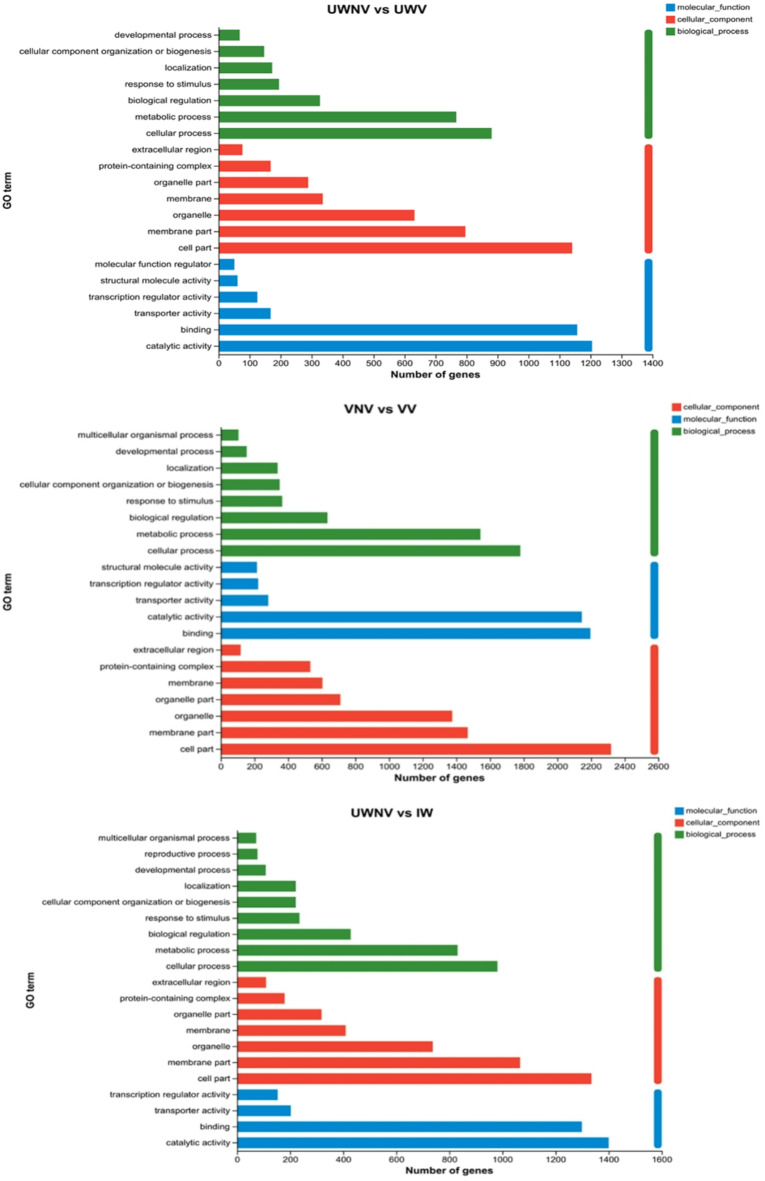
GO annotation histogram of differently expressed genes.

GO was widely utilized in bioinformatics for categorizing gene functions, and it categorized them into three main areas: Biological Process, Cellular Component, and Molecular Function. Analysis revealed that genes associated with cellular and metabolic activities were predominant in the Biological Process category. Within the Cellular Component realm, genes related to cellular structures and membrane components were most frequently identified. In the Molecular Function domain, genes engaged in binding and catalytic processes were found to be the most numerous.However, in the comparisons of UWNV vs. UWV and UWNV vs. IW, catalytic activity was more predominant than binding, while in the comparison of VNV vs. VV, binding was more predominant than catalytic activity (Fig. 5).

### Differential gene expression GO enrichment analysis

**Fig. 6:**
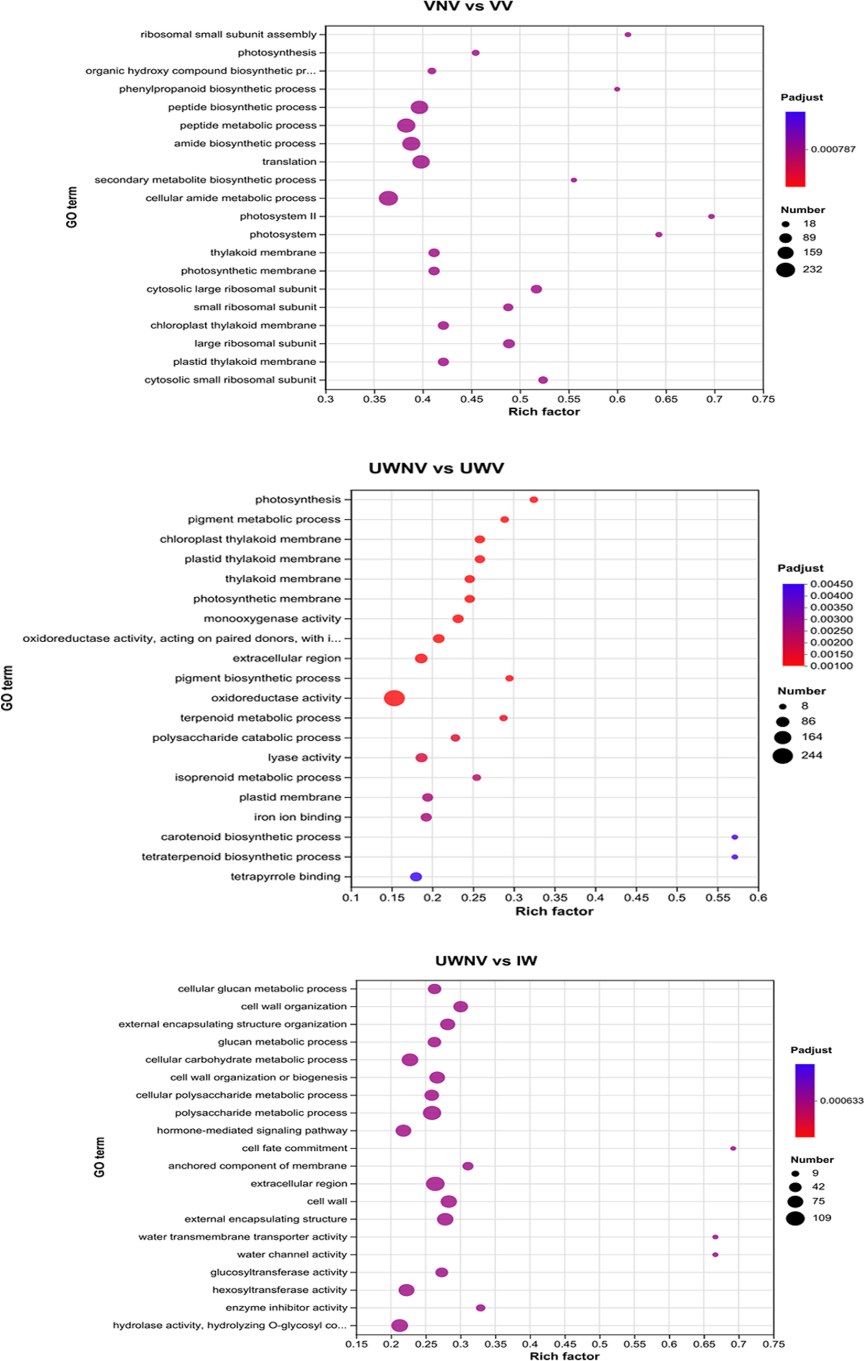
GO enrichment bubble diagram of differently expressed genes.

It was observed that the top 20 most significantly enriched GO terms after GO enrichment analysis for each group. In the comparison of VNV vs. VV, the most significantly enriched term was the cellular amide metabolic process in the molecular function category, with a total of 232 genes. In the comparison of UWNV vs. UWV, the enrichment of oxidoreductase activity was the most significant, with a total of 244 genes. In the comparison of UWNV vs. IW, the enrichment of the extracellular region was the most significant, with a total of 109 genes (Fig. 6).

### Differential gene expression KEGG enrichment analysis

**Fig. 7:**
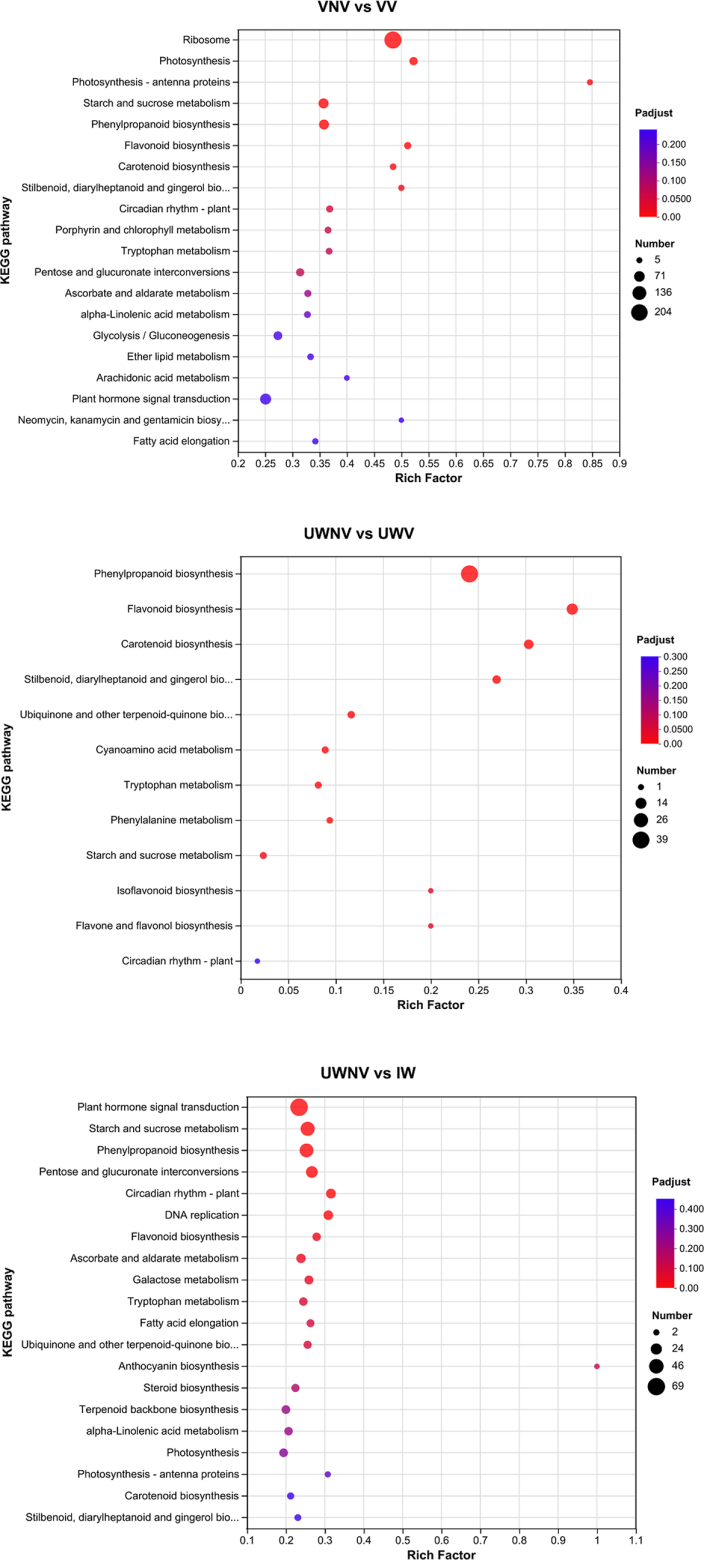
KEGG enrichment bubble diagram of differently expressed genes.

It was observed that the top 20 most significantly enriched KEGG terms after KEGG enrichment analysis for each group (Fig. 7). The bubble chart displayed the top 20 pathways enriched by the DEGs groups of VNV vs. VV, UWNV vs. UWV, and UWNV vs. IW. Among them, genes in the ‘Flavonoid biosynthesis’ and ‘Carotenoid biosynthesis’ pathways may have been involved in the synthesis and accumulation of anthocyanins in the petals of *I. morsei*.

It was observed that among the significantly enriched Pathways in the comparison of VNV vs. VV, there were 22 unigenes involved in the flavonoid biosynthesis pathway, with 8 of them being DEGs; and there were 16 unigenes involved in the carotenoid biosynthesis pathway, with 12 of them being DEGs. In the significantly enriched Pathways of the UWNV vs. UWV comparison, there were 15 unigenes involved in the flavonoid biosynthesis pathway, with 9 of them being DEGs; and there were 10 unigenes involved in the carotenoid biosynthesis pathway, with 9 of them being DEGs. In the significantly enriched Pathways of the UWNV vs. IW comparison, there were 12 unigenes involved in the flavonoid biosynthesis pathway, with 8 of them being DEGs; and there were 7 unigenes involved in the carotenoid biosynthesis pathway, with 4 of them being DEGs (Table S2). From the above, it was known that the flavonoid biosynthesis pathway was significantly enriched in the pairwise comparisons between the spotted and non-spotted areas of the petals of *I. morsei*, indicating that flavonoid biosynthesis was active in the process of flower color formation in *I. morsei*.

By comparing the DEGs with biosynthetic pathways, a biosynthetic pathway map of the DEGs was obtained. In the comparison of VNV vs. VV, within the flavonoid biosynthesis pathway, the expressions of CYP73A, F3H, FLSS, and DFR were upregulated, while the expressions of CYP75A, ANS, and ANR were downregulated, and CHS showed both upregulation and downregulation in expression (Figure S5). In the carotenoid biosynthesis pathway, the expressions of PDS, Z-ISO, crtISO, LUT5, crtZ, LUT1, *CCS*, NCED, and CYP707A were upregulated. The expression of ABA1 was downregulated, and the expressions of crtB and ZEP showed both upregulation and downregulation (Figure S6).

In the comparison of UWNV vs. UWV, within the flavonoid biosynthesis pathway, the expressions of CHS and DFR were upregulated, while the expressions of CYP73A, F3H, FLSS, CYP75A, ANS, ANR, and LAR were downregulated (Figure S7). In the carotenoid biosynthesis pathway, the expressions of crtB, PDS, Z-ISO, crtISO, crtZ, LUT1, CCS, ZEP, and CYP707A were upregulated (Figure S8).

In the comparison of UWNV vs. IW, within the flavonoid biosynthesis pathway, the expressions of CHS, DFR, and ANR were upregulated, while the expressions of CYP73A, F3H, FLSS, CYP75B1, and LAR were downregulated (Figure S9). In the carotenoid biosynthesis pathway, the expressions of crtB, ABA1, and CYP707A were upregulated, while the expression of NCED was downregulated (Figure S10).

### Differential gene expression analysis

In the transcriptome analysis of *I morsei*, we conducted a detailed classification and functional annotation of differentially expressed genes (DEGs). Using the NCBI Conserved Domain Database (CDD), we performed a conserved domain search on the DEGs to identify their gene families. In this study, the most annotations were related to *PAL* structural genes and MYB transcription factors (Table S3). The majority of these genes encoded proteins belonging to the the SANT super family and Myb transcription factor family, which play important roles in the regulation of anthocyanin biosynthesis.

### Differential expression analysis of *PAL* genes

**Fig. 8:**
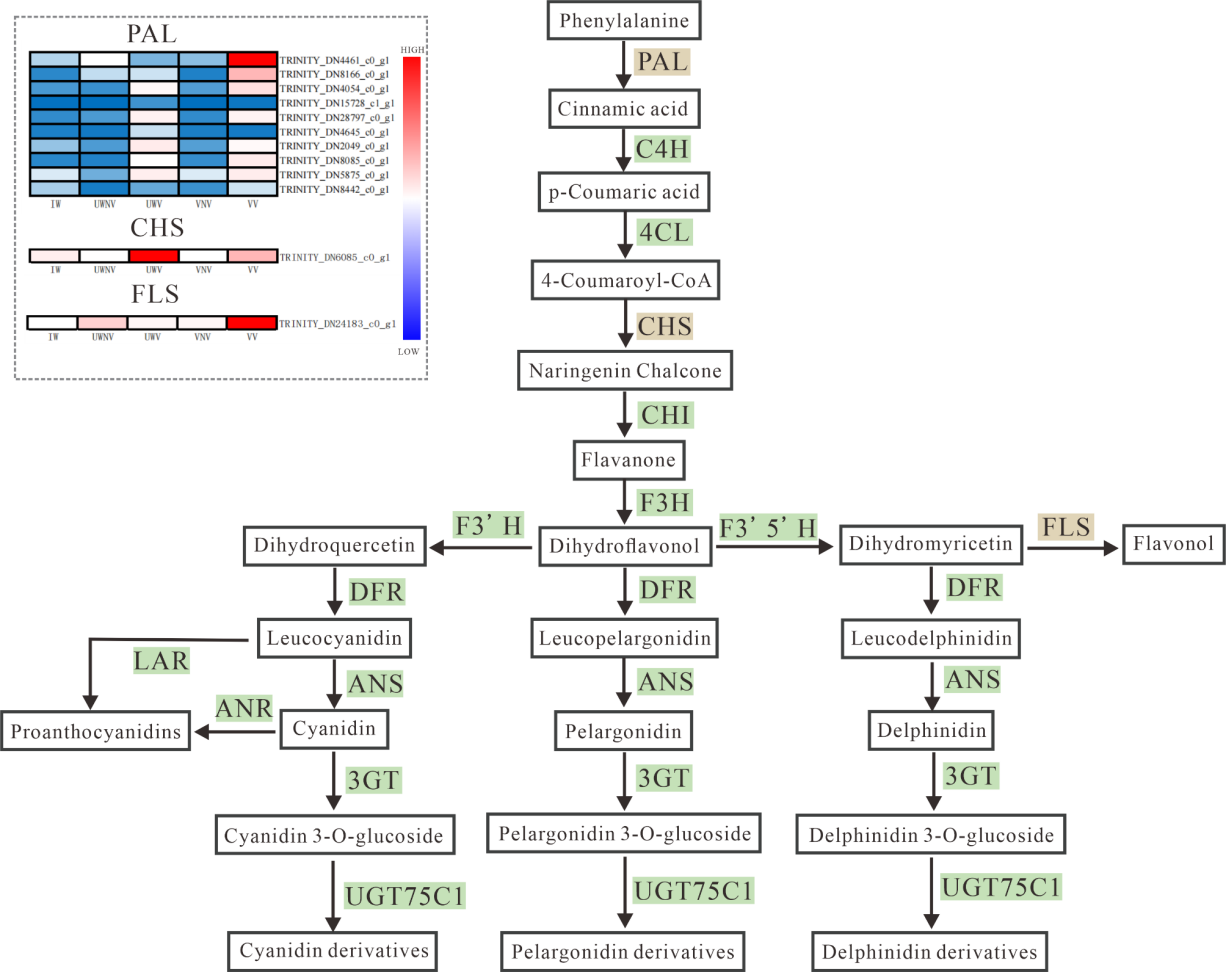
Expression patterns of key genes in the anthocyanin biosynthesis pathway in five different coloration regions (VV, VNV, UWV, UWNV, and IW) of I. morsei.The progression of the color scale from red to blue represents the levels of gene expression.

**Fig. 9:**
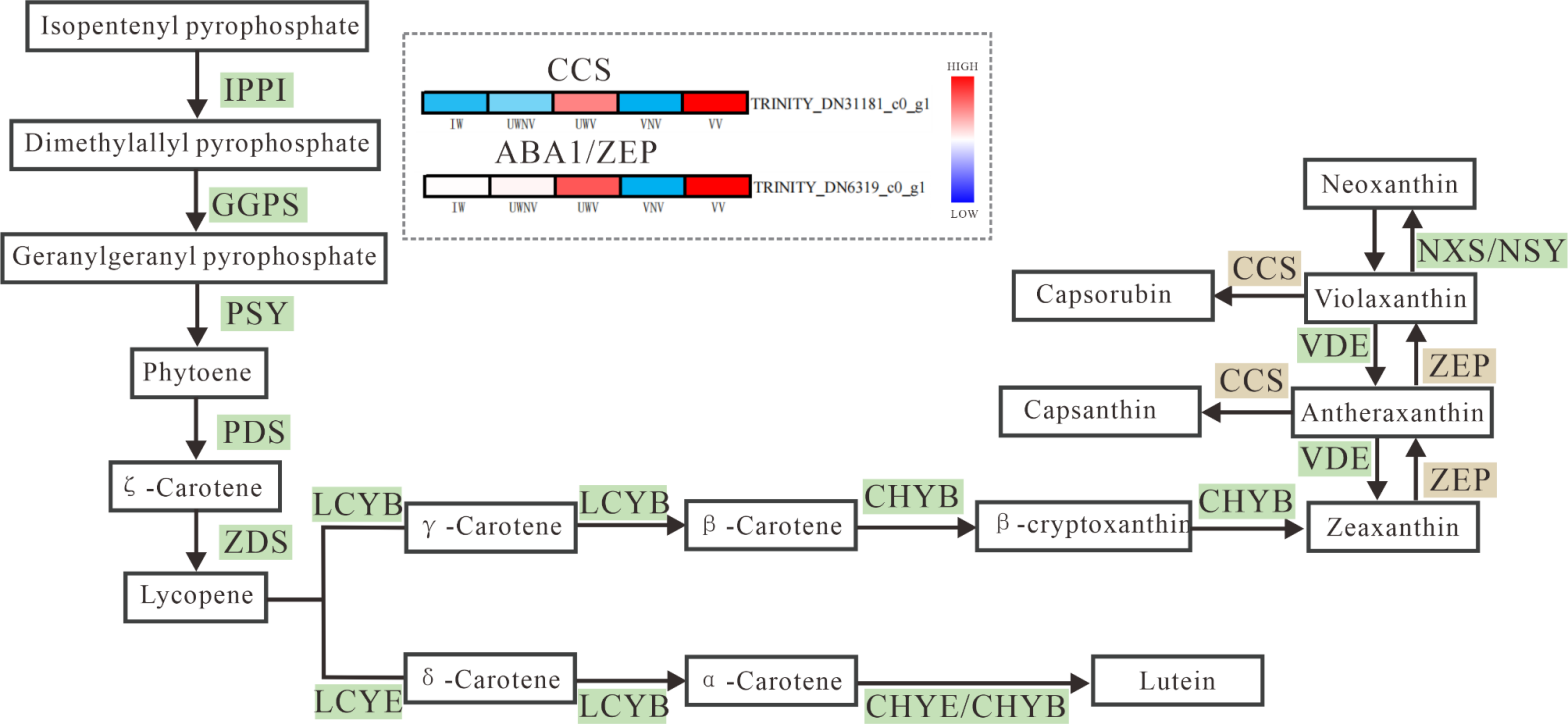
Expression patterns of key genes in the carotenoid biosynthesis pathway in the five different coloration regions (VV, VNV, UWV, UWNV, and IW) of. *I.* morsei.The progression of the color scale from red to blue represents the levels of gene expression.

In this study, we constructed simplified diagrams of the anthocyanin biosynthesis pathway (Fig. 8 and the carotenoid biosynthesis pathway (Fig. 9). By analyzing the transcriptome data of *I. morsei*, we identified 10 differentially expressed genes (DEGs) related to the *PAL* gene and predicted the molecular mechanisms leading to variegation in *I. morsei* using a clustered heatmap.

In the comparison of VV vs. VNV, there were a total of 2 differentially expressed genes (Figure S11). It was observed from the clustered heatmap that the branches of TRINITY_DN4461_c0_g1 and TRINITY_DN8166_c0_g1 were closely related, suggesting that their expression patterns might also be similar. Furthermore, these genes had higher expression levels in VV and lower in VNV, leading to the further speculation that they might have a positive regulatory effect in the formation of VV.

In the comparison of UWV vs. UWNV, there were a total of 8 differentially expressed genes (Figure S12). It was observed from the clustered heatmap that the branches of TRINITY_DN4054_c0_g1, TRINITY_DN15728_c1_g1, TRINITY_DN28797_c0_g1, TRINITY_DN4645_c0_g1, TRINITY_DN2049_c0_g1, and TRINITY_DN8085_c0_g1 were closely related, suggesting that their expression patterns might also be similar. And the expression levels of these six differentially expressed genes, along with TRINITY_DN5875_c0_g1, were higher in UWV and lower in UWNV. It was hypothesized that these genes might have a positive regulatory role in the formation of UWV. Additionally, the expression level of TRINITY_DN8166_c0_g1 was lower in UWV and higher in UWNV, leading to the speculation that this gene might have a negative regulatory effect in the formation of UWV.

In the comparison of IW vs. UWNV, there were a total of 2 differentially expressed genes (Figure S13). It was observed from the clustered heatmap that the branches of TRINITY_DN8166_c0_g1 and TRINITY_DN8442_c0_g1 were distant from each other, suggesting that their expression patterns might be quite different. Furthermore, TRINITY_DN8166_c0_g1 had lower expression in IW and higher in UWNV, leading to the hypothesis that this gene might have a positive regulatory role in the formation of UWNV. Conversely, TRINITY_DN8442_c0_g1 exhibited higher expression in IW and lower in UWNV, speculating that this gene might have a negative regulatory effect in the formation of UWNV.

### Differential expression analysis of MYB-related transcription factor genes

In the biosynthesis of anthocyanins, it is regulated by many transcription factors.In this study, we identified TFs related to anthocyanin biosynthesis in *I. morsei*. We identified 4 key differentially expressed TFs related to MYB in the comparison of VV vs. VNV, 5 in UWV vs. UWNV, and 13 in UWNV vs. IW.

In the comparison of VV vs. VNV, there were a total of 4 differentially expressed genes (Figure S14). It was observed from the clustered heatmap that the branches of TRINITY_DN1270_c0_g1 and TRINITY_DN17104_c0_g1 were closely related, as were the branches of TRINITY_DN940_c0_g1 and TRINITY_DN8574_c0_g2, suggesting that their expression patterns might also be similar. Additionally, these genes were found to have higher expression levels in VV, and it was speculated that they might play a positive regulatory role in the formation of VV flower color.

In the comparison of UWV vs. UWNV, there were a total of 5 differentially expressed genes (Figure S15). It was observed from clustered dendrogram that the branches of TRINITY_DN1275_c0_g1, TRINITY_DN4179_c0_g1, TRINITY_DN10819_c0_g1, and TRINITY_DN12273_c0_g1 were closely related, suggesting that their expression patterns might also be similar. These genes exhibited higher expression levels in UWNV and lower in UWV, leading to the speculation that they might have a negative regulatory effect on the formation of UWV. Additionally, TRINITY_DN717_c0_g1 showed higher expression in UWV and lower in UWNV, suggesting that this gene might have a positive regulatory role in the formation of UWV.

In the comparison of IW vs. UWNV, there were a total of 13 differentially expressed genes (Figure S16). It was observed from the clustered heatmap that the branches of TRINITY_DN4620_c0_g1, TRINITY_DN4858_c0_g1, TRINITY_DN14856_c0_g1, TRINITY_DN11987_c0_g5, TRINITY_DN11935_c0_g1, TRINITY_DN3568_c0_g1, TRINITY_DN3152_c0_g2, TRINITY_DN24308_c0_g1, and TRINITY_DN717_c0_g1 were closely related, suggesting that their expression patterns might also be similar. These genes had higher expression levels in IW and lower in UWNV, leading to the speculation that they might have a negative regulatory effect on the formation of UWNV. Additionally, the branches of TRINITY_DN19979_c0_g2 and TRINITY_DN8574_c0_g1 were closely related, as were the branches of TRINITY_DN17104_c0_g1 and TRINITY_DN3971_c0_g2. It was speculated that the expression patterns among these four genes might also be similar, and all of these differentially expressed genes had lower expression in IW and higher in UWNV. It was hypothesized that these four genes might have a positive regulatory role in the formation of UWNV.

### Flavonoid and carotenoid biosynthetic pathway differential expression gene screening

This study utilized transcriptome data to analyze the expression patterns of genes involved in the flavonoid and carotenoid biosynthetic pathways, and identified six key genes that regulate the flavonoid biosynthesis pathway, including four structural genes (*PAL*, *PAL*3, *CHS1*, *FLS*) (Fig. 8) and two regulatory genes (CPC, MSI4). Additionally, two key genes that regulate the carotenoid biosynthesis pathway were selected, both being structural genes (*ABA1*, *CCS*) (Fig. 9), and all were found to have higher expression levels in the petal spotted area of *I. morsei* (Table S4).

### Transcriptome data qRT-PCR validation

**Fig. 10:**
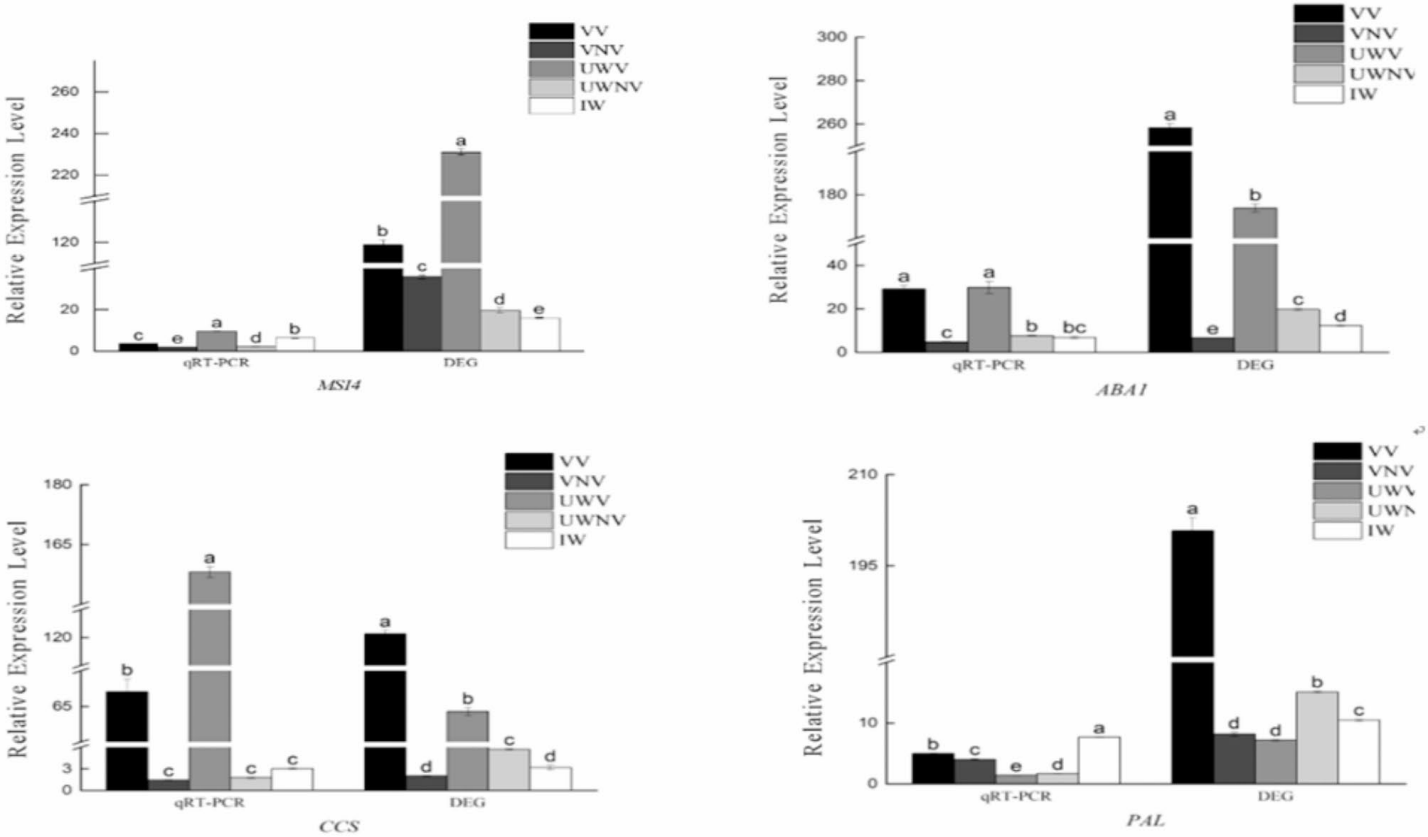
qRT-PCR validation of transcriptome data.

To verify the reliability of the transcriptome data and the speculations regarding the reasons for pigment accumulation in the spotted and non-spotted areas of the *I. morsei*, this study selected one regulatory gene (MSI4) and three structural genes (*ABA1*, *PAL*, *CCS*) related to the formation of flower spots for qRT-PCR analysis. The analysis results showed that the expression trends of the four genes from qRT-PCR were basically consistent with those in the transcriptome data. The MSI4 and *PAL* genes showed significant differences in expression across the five distinct coloring regions of *I. morsei*. The *ABA1* gene exhibited higher expression in the vexil spotted area and the upper wing petal spotted area, with similar levels of expression; whereas, it had lower expression in the vexil non-spotted area, the upper wing petal non-spotted area, and the lower wing petal, with relatively similar levels of expression. The *CCS* gene showed significant differences in expression in the vexil spotted area and the upper wing petal spotted area of *I. morsei*. In contrast, its expression levels were similar and relatively lower in the vexil non-spotted area, the upper wing petal non-spotted area, and the lower wing petal. (Fig. 10). This demonstrated that the transcriptome data we measured and the results of the qRT-PCR analysis were relatively accurate and reliable, further indicating that the structural and regulatory genes related to the regulation of pigment accumulation in different zones of the *I. morsei* petals, which were screened in this study, had credibility.

## Discussion

This study conducted transcriptome sequencing on five distinct coloring regions of the spotted and non-spotted areas of the *I. morsei* petals, aiming to identify genes related to the formation of flower spots. The goal was to uncover candidate genes that play a key role in the formation of flower spots in *I. morsei* and to further explore the regulatory mechanisms of the flavonoid and carotenoid biosynthesis pathways in this species. The results showed that the Q30 values of the five sequenced samples exceeded 94%, which was superior to the 90% achieved by Peng in handling *Anthurium andraeanum*, the 88. 49% by Wang in handling *Narcissus tazettasubsp. chinensis*, the 81. 92% and 82. 4% by Xu in handling the *Iris halophila* and *Iris halophila var. Sogdiana* and the 93. 43% by Guo in handling *Sophora japonica*^42–45^. This indicated that the sequencing quality of this study was high, and its results were reliable and accurate. This study assembled a total of 39,917 unigene sequences, which was higher than the 36,006 unigene sequences processed by from the stem scales of *Narcissus tazetta L. var*, but lower than the 86,195 unigene sequences processed by Su from *Paeonia suffruticosa* and *Paeonia lactiflora*, and the 41,672 unigene sequences processed by Xiang from the stem of *Xanthoceras sorbifolium*. This study obtained an average unigene length of 1115. 26 bp, which was higher than the 589. 1 bp processed by Chen et al. (2021) from *Batrachium bungei*, but lower than the 1189 bp processed by XiaoDi Su (2019) from *Paeonia suffruticosa* and *Paeonia lactiflora*, and the 1303 bp processed by Xiang (2019) from the *Xanthoceras sorbifolium*^46–49^.

Through KEGG enrichment analysis, it was determined that the differences in flower spot formation in *I. morsei* were primarily enriched in the “flavonoid synthesis pathway” and the “carotenoid synthesis pathway”. Further speculation suggested that variations in these pathways, the “flavonoid synthesis pathway” and the “carotenoid synthesis pathway”, might have an impact on the formation of flower spots in *I. morsei*. Zhou et al. found that the flavonoid metabolic pathway played a central role in the coloring of *Camellia nitidissima*^50^. Li et al. discovered that the purple coloration of *Salvia miltiorrhiza* petals was associated with differential genes in the flavonoid synthesis pathway^51^. Xia et al. conducted research and found that the upregulation of carotenoid concentration and the genes related to carotenoid biosynthesis primarily promoted the color transformation of the petals in *Lonicera japonica Thunb*^52^. These studies further validated that the enriched differential genes in the flavonoid synthesis pathway and the carotenoid synthesis pathway of *I. morsei* could be the cause of the flower spot formation in *I. morsei* .

Additionally, the analysis discovered key genes in the flavonoid biosynthesis of *I. morsei*, which included two regulatory genes, CPC and MSI4, and four structural genes, *PAL*, *PAL*3, *CHS1*, and *FLS*. A total of two key genes were identified for the regulation of carotenoid biosynthesis, both being structural genes, *ABA1* and *CCS*. To date, there are many transcription factors involved in the regulation of anthocyanin synthesis in plants, but the ones that are relatively well-studied include MYB, bHLH, and WD40. These factors can bind to each other to form a complex, working together in the regulation of anthocyanins^53,54^. MYB was recognized as the largest transcription factor family in plants, and was divided into R3-MYB, R2R3-MYB, and R1R2R3-MYB due to the different numbers of structural domains. The R2R3-MYB transcription factors were known to play a very significant role in the regulation of anthocyanin biosynthesis, being capable of exerting both positive and negative regulatory effects.

CPC, as a MYB transcription factor in the MYB domain, was found to repress the biosynthesis of anthocyanins by regulating the expression of structural genes^55^. Zhang found that *Nicotiana tabacum* L. was utilized as an experimental subject, and an overexpression vector of the *AtCPC* gene was introduced into it^56^ It was observed that the flower color of the *Nicotiana tabacum* L. turned lighter or whiter. Gong et al. discovered through experiments that the expression of the WD40 transcription factor HmWDR68 in the blue sterile flowers of the *Hydrangea macrophylla* ‘forever summer’ was significantly higher than in other tissues and organs^57^. Furthermore, compared with *Hydrangea macrophylla* varieties of different flower colors sterile flowers the expression of HmWDR68 in the blue sterile flowers was markedly elevated. In this study, MSI4 was found to have significant differences in expression between the spotted and non-spotted areas of the *I. morsei*, with higher expression in the spotted areas. CPC and MSI4 are respectively part of the MYB and WD40 transcription factors, and it is speculated that their functions are similar to those of the two.

Lepelley et al. found that *PAL* played a key role in the accumulation of flavonoid compounds^58^. Wang discovered that when an RNA interference vector targeting the *PAL* gene was introduced into the *Malus* ‘Starkimson’, there was a decrease in anthocyanin content^59^. In 2016, Yang found that the leaf change period of the *Pyrus calleryana*, the activity of PAL was negatively correlated with anthocyanin^60^. In this study, *PAL* was found to have significantly higher expression in the variegated areas of the anterior petal of the *I. morsei* compared to the non-variegated areas, while in the variegated areas of the wing petals, its expression was significantly lower than in the non-variegated areas.It was speculated that the *PAL* gene might have an important regulatory effect on the formation of flower spots in *I. morsei*. Liang discovered that *CHS* was involved to a certain extent in regulating the synthesis of anthocyanins in the petals of *Red-Flowered Strawberry*^61^. Yuka Ohta et al. found that post-transcriptional gene silencing of CHS leads to the formation of striped bicoloration in *Japanese gentian*^62^. From this, it can be speculated that the *CHS* gene may play an important role in the formation of spots in *I. morsei*. Liu et al.^63^. conducted research and discovered that Ma*FLS* played a key role in flavonol biosynthesis and flower coloration in *Grape Hyacinth.* The gene was primarily expressed during the early stages of flower development in *Grape Hyacinth*. Heterologous expression of Ma*FLS* in *Oncidium hybridum* demonstrated a significant reduction in anthocyanin content and an increase in flavonol accumulation, leading to a decrease in pigmentation of the petals. From this, it can be further speculated that this gene plays an important role in the flavonoid synthesis pathway of *I. morsei*. Nakkanong et al.^64^ found in their research on *Cucurbita moschata* that *ZEP* has a regulatory effect on the production of violaxanthin and lutein. Chiou et al.^65^ discovered that in the petals of the orange *Oncidium hybridum*, a reduction in the expression level of ZEP might promote the β-carotene *FLS* gene, which has a certain regulatory effect on the white coloration of the flower.In this study, the *ABA1* gene (also known as the *ZEP* gene) was found to be highly expressed in the spotted areas of the vexilla variegation and upper wing variegation of the *I. morsei*, with significant differences in expression between the spotted and non-spotted areas. This further demonstrates that the ABA1 gene plays a regulatory role in the formation of *I. morsei* variegation. Zhang^66^ discovered that *CCS* possesses the dual function of catalyzing the conversion of antheraxanthin to capsanthin/capsorubin and the conversion of lutein to capsanthin, which can confer a red color to the fruits or flowers of certain plants. Additionally, *CCS* was found to have high expression in the spotted areas of the vexilla variegation and upper wing variegation of the *I. morsei*, confirming that this gene has a positive regulatory effect on the formation of variegated patterns in the *I. morsei*.

## Materials and methods

### Plant materials

The plant material used in this study is not a rare or endangered species. Samples were collected from wild populations outside of protected areas and are cultivated and preserved at the experimental base of Southwest Forestry University without the need for permits/licenses. The voucher specimen is deposited in the Guangxi Botanical Garden Herbarium (IBK), identified by Yang Ping and others, with the deposition number: IBK00428295, which flower composition mainly consisted of the vexil, upper wing petal, sepal, and lower wing petal (Fig. 1a). During the peak blooming period, five distinct coloring regions were sampled from petals that were in good growing condition and free of diseases and pests (the vexilla variegation, the vexilla non-variegation; the upper wing variegation; the upper wing non-variegation; the: inferolateral wing,) (Fig. 1b). After being separated from the plant, the samples were quickly placed into liquid nitrogen and properly labeled, then stored in an ultra-low temperature freezer at −80℃ in preparation for transcriptome sequencing. This study was based on high-throughput second-generation sequencing methods, using the Illumina Novaseq 6000 sequencing platform (sequencing work was carried out by Majorbio) for transcriptome sequencing.

### Transcriptome sequencing and analysis

#### RNA extraction and detection

Total RNA from the five distinct coloring regions of the *I. morsei* during the peak blooming period was extracted according to the instructions of the Omega Company’s plant RNA extraction kit and was stored at −80℃ in an ultra-low temperature freezer. Agarose gel electrophoresis was used to check the integrity of the RNA, and nucleicacid protein instrument was used to measure the optical densitys of the RNA, thereby determining whether the concentration and purity of the RNA met the requirements of the experiment.

### Transcriptome sequencing and assembly

The construction of the cDNA library was carried out using the Illumina NEB Next^®^ Ultra™ RNA Library Prep, which yielded Raw data. Subsequent quality control was performed to obtain clean data, and de novo assembly was conducted to generate non-redundant unigenes. In this study, we sequenced the five distinct coloring regions and assembled the transcriptomes of the spotted and non-spotted areas of the *I. morsei.*

### Sequence analysis and annotation

Following the offloading of sequencing data, the nucleotide sequences of unigenes in *I. morsei* had to be assembled after undergoing a series of processes including quality control, filtering, and assembly.

To achieve a comprehensive functional annotation of unigene this research employed the comparison software BLASTX(BasicLocal Alignment Search Tool)(E-value ≤ 1. E-5)to compare the unigene sequences with the open databases, this allows for the functional annotation and classification of the sequences. The public alignment databases include GO (Gene Ontology), KEGG (Kyoto Encyclopedia of Gene sand Genomes, http://www.genome.jp/kegg), Swiss-Prot (A manually annotated and reviewed protein sequence database, http://www.genome.jp/kegg), and the Unigene Sequence Search Tool (Evalue ≤ 1. E-5). and reviewed protein sequence database, http://www.ebi.ac.uk/uniprot/), COG/KOG (EuKaryotic Orthologous Group,http://ftp.ncbi.nih.gov/pub/COG/KOG), eggNOG (evolutionary genealogy of genes: Non-supervised Orthologous Groups, http://eggnogdb.embl.de/), Pfam (a data base of conserved Protein families ordomais), and NR (NCBI non-redundant proteins). non-redundant protein, https://blast.ncbi.nlm.nih. gov-/Blast. cgi) databases were compared (E- value ≤ 1. 0E-05)^67^.

### Screening and enrichment analysis of differentially expressed genes (DEGs)

Assemble the sequences and then perform a BLASTx comparison with the protein database. Based on the comparison results, use the software RSEM to quantify the gene expression levels. Subsequently, standardize the gene expression levels using the FPKM method, and then screen for differentially expressed genes based on the expression levels.

### MSI4, *ABA1*, *FLS*, *PAL3*, *CCS*, CPC and *CHS1* genes expression and analysis

Primers were designed based on the sequences of MSI4, *ABA1*, *FLS*, *PAL*3, *CCS*, CPC, and *CHS1*, and *IuActin* was used as an internal reference gene. The expression differences of MSI4, *ABA1*, *FLS*, *PAL3*, *CCS*, CPC, and *CHS1* were analyzed by qRT-PCR on the full bloom samples of five distinct coloring regions of *I. morsei*. qRT-PCR was carried out on Roche: LightCycler^®^ 480 II Fluorescent Quantitative PCR Instrument. qRT-PCR reaction system (20 µL): qPCR SYBR Green Master Mix 10 µL, ddH2O 6 µL, forward and reverse primers each 0. 5 µL, template cDNA 3 µL. The specific program was as follows: pre-denaturation at 95℃ for 15 s; denaturation at 60℃ for 30 s, annealing at 72℃ for 1 min, 40 cycles. Based on the qRT-PCR results, the Status values of each set of data were recorded. The significance of the differences in the measured data was analyzed using IBM SPSS Statistics 25 software (*P* < 0. 05), and finally, the expression levels of seven genes, including MSI4, *ABA1*, *FLS*, *PAL3*, *CCS*, CPC, and *CHS1*, were plotted using Origin 2022 software.

## Conclusion

Through transcriptome sequencing, 39,917 unigene sequences were obtained, along with 39,110 differentially expressed genes, with the number of downregulated genes exceeding that of upregulated ones.Three key genes in the flavonoid biosynthetic pathway, *FLS*, *PAL*, and *CHS1*, were screened out. Additionally, two key genes in the carotenoid biosynthetic pathway, *ABA1* and *CCS*, were identified. Furthermore, two regulatory genes, MSI4 and CPC, were also selected.

This study selected five distinct coloring regions (vexil spotted area, vexil non-spotted area, upper wing petal spotted area, upper wing petal non-spotted area, and lower wing petal) from the petals of *I. morsei* with good growth condition and free from diseases and pests during the peak blooming period as materials for transcriptome sequencing. It explored the synthetic mechanism of flower spots in *I. morsei* from the perspective of molecular biology, providing a certain basis of data and theoretical support for further research on the formation of flower spots, flower color improvement, and the cultivation of new varieties of *Impatiens*.

## Electronic supplementary material

Below is the link to the electronic supplementary material.

Supplementary Material 1

Supplementary Material 2

## Data Availability

Raw data have been deposited to National Center for Biotechnology Information (NCBI) under the BioProject number PRJNA1196802.
