# Supplementary material for: Transcriptome analysis reveals the expression of genes involved in the formation of petal variegation in Impatiens morsei
Source: Sci Rep. 2025 Apr 2;15:11265. doi: 10.1038/s41598-025-93846-0 (PMC11965560; doi:10.1038/s41598-025-93846-0)
Supplement: Supplementary file 2 — Supplementary Material 2 [file 41598_2025_93846_MOESM2_ESM.docx]

**Transcriptome analysis reveals the expression of genes involved in the formation of petal variegation in *Impatiens morsei*.**

Hai-Ge Liu^1,2,+^  Ting-Ting Feng^1,3,+^ Si-Yu Ren^1,4,+^ Jian-Yuan Yan^1^  Lu-Qiu Zhao^1^  Xiao-Li Zhang^1^  Mei-Juan Huang^1,2,^*& Hai-Quan Huang^[[1]](#footnote-0),4,^*

^1^College of Landscape Architecture and Horticulture Sciences, Southwest Forestry University, Yunnan, 650224, China.. ^2^Southwest Research Center for Engineering Technology of Landscape Architecture(State Forestry and Grassland Administration),Yunnan, 650224, China. ^3^Yunnan Engineering Research Center for Functional Flower Resources and Industrialization,Yunnan, 650224, China. ^4^Research and Development Center of Landscape Plants and Horticulture Flowers, Yunnan, 650224, China.

**Supplementary Figures**

**Fig. S1 Unigene GO annotation diagram**

**Fig. S2 Classified statistical chart of pathway**

**Fig. S3 COG classification statistics**

**Fig. S4 NR annotated species distribution map**

**Fig. S5 Flavonoid biosynthesis pathway in VNV vs VV^1,2,3^.**

**Fig. S6 Carotenoid biosynthesis pathway in VNV vs VV^1,2,3^.**

**Fig. S7 Flavonoid biosynthesis pathway in UWNV vs UWV^1,2,3^.**

**Fig. S8 Carotenoid biosynthesis pathway in UWNV vs UWV^1,2,3^.**

**Fig. S9 Flavonoid biosynthetic pathways in UWNV vs IW^1,2,3^.**

**Fig. S10 Carotenoid biosynthesis pathway in UWNV vs IW^1,2,3^.** Green underlining indicates a change, no underlining indicates no change. A red wireframe indicates an upward adjustment and a blue wireframe indicates a downward adjustment.

**Fig. S11 Differential expression and comparison of MYB in VV and VNV Unigenes.**

**Fig. S12 Differential expression and comparison of MYB in UWV and UWNV Unigenes.**

**Fig. S13 Differential expression and comparison of MYB in IW and UWNV Unigenes.**

**Fig. S14 Differential expression and comparison of PAL in VV and VNV Unigenes.**

**Fig. S15 Differential expression and comparison of PAL in UWV and UWNV Unigenes.**

**Fig. S16 Differential expression and comparison of PAL in IW and UWNV Unigenes.**

**Figure. S1**


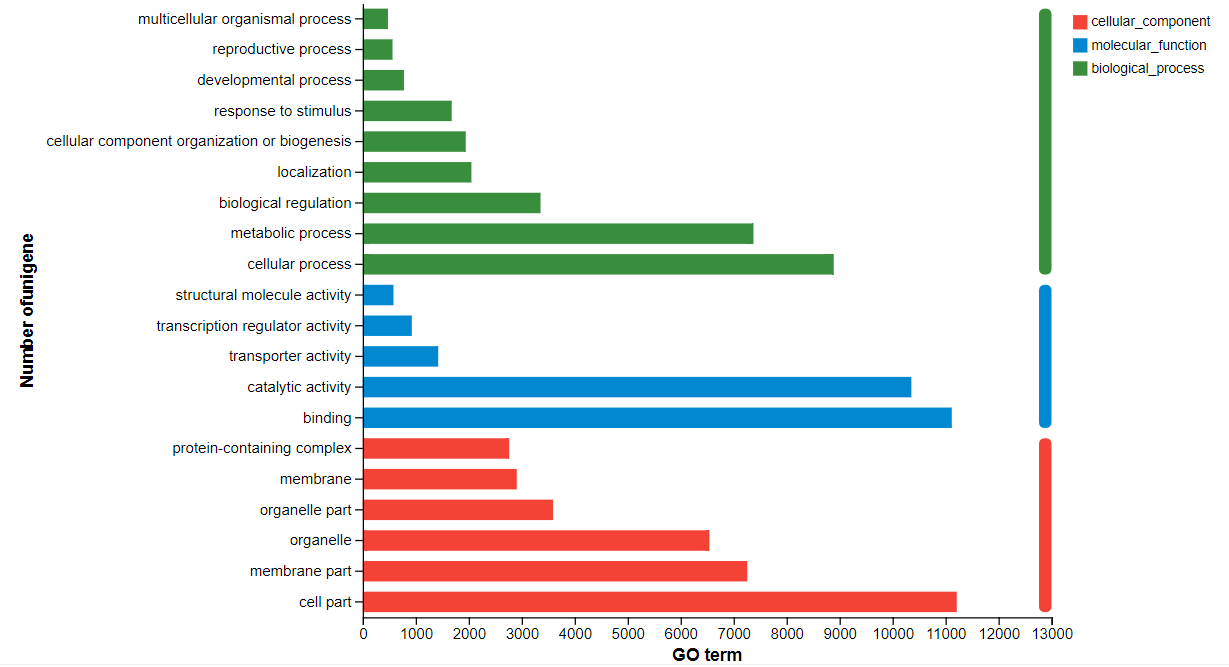
**Figure. S2**


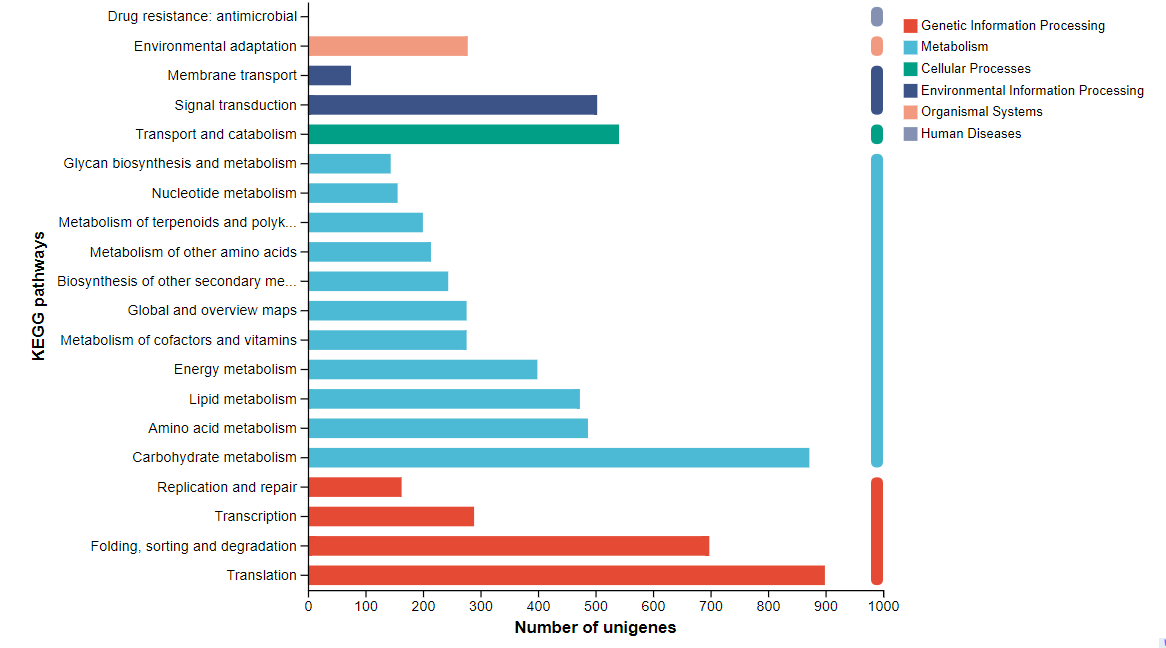


**Figure. S3**


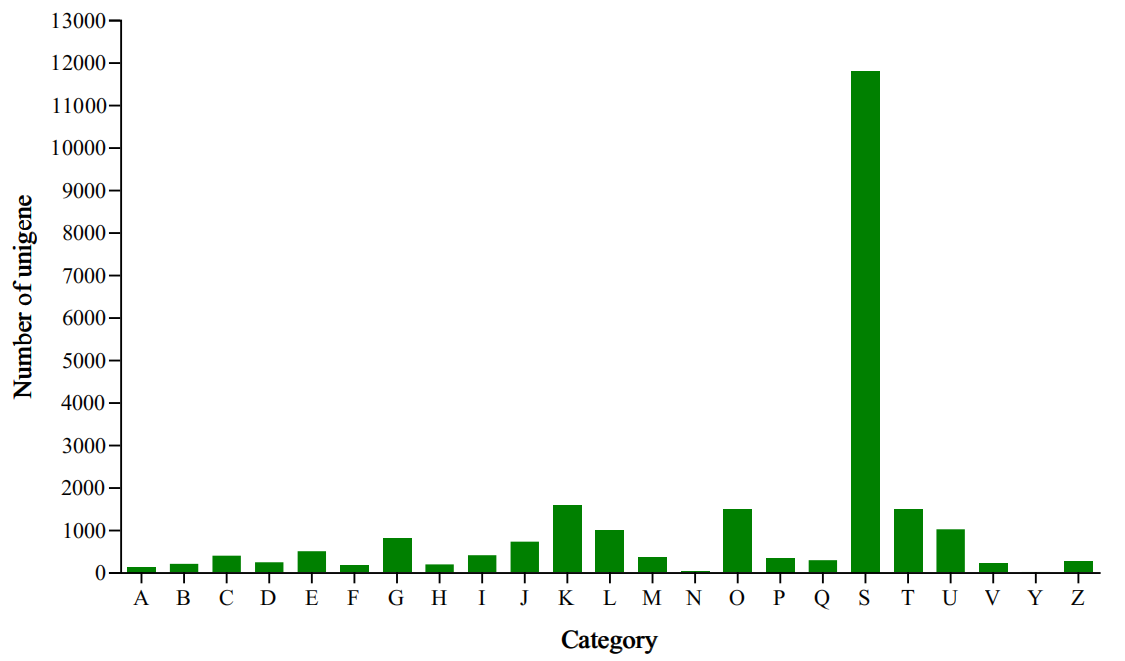

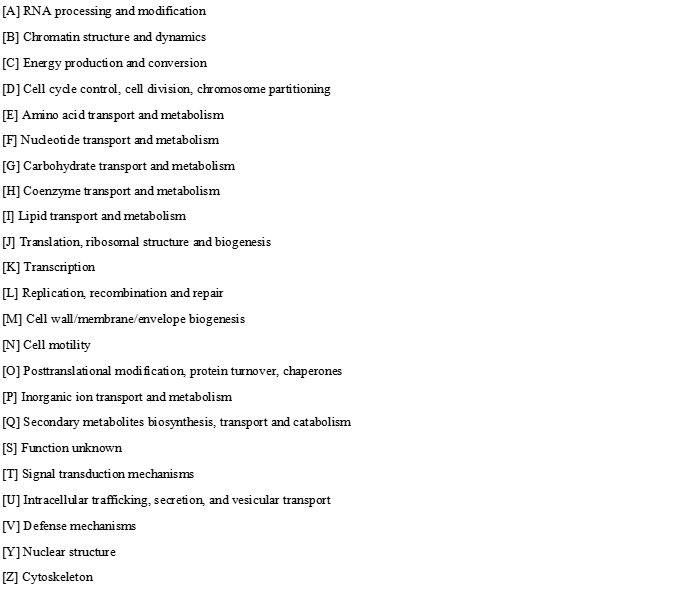
**Figure. S4**


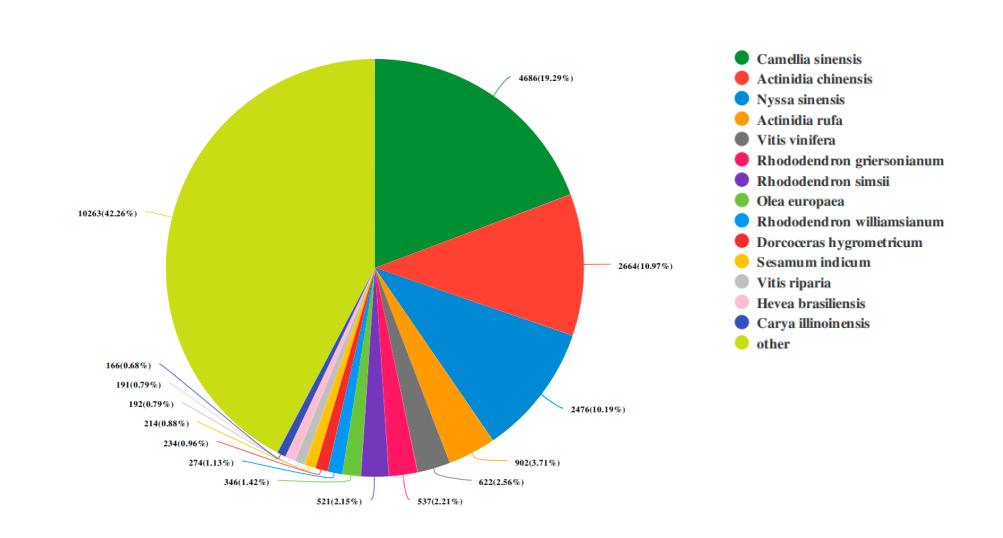


**Figure. S5**


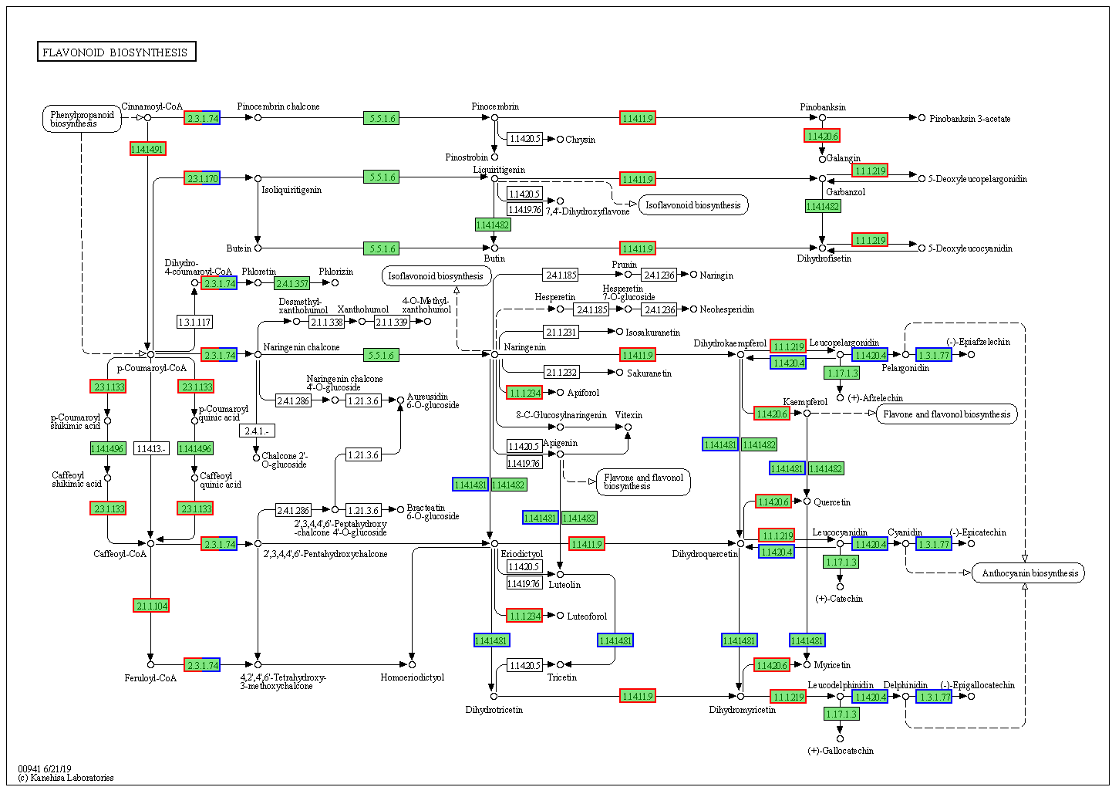


**Figure. S6**


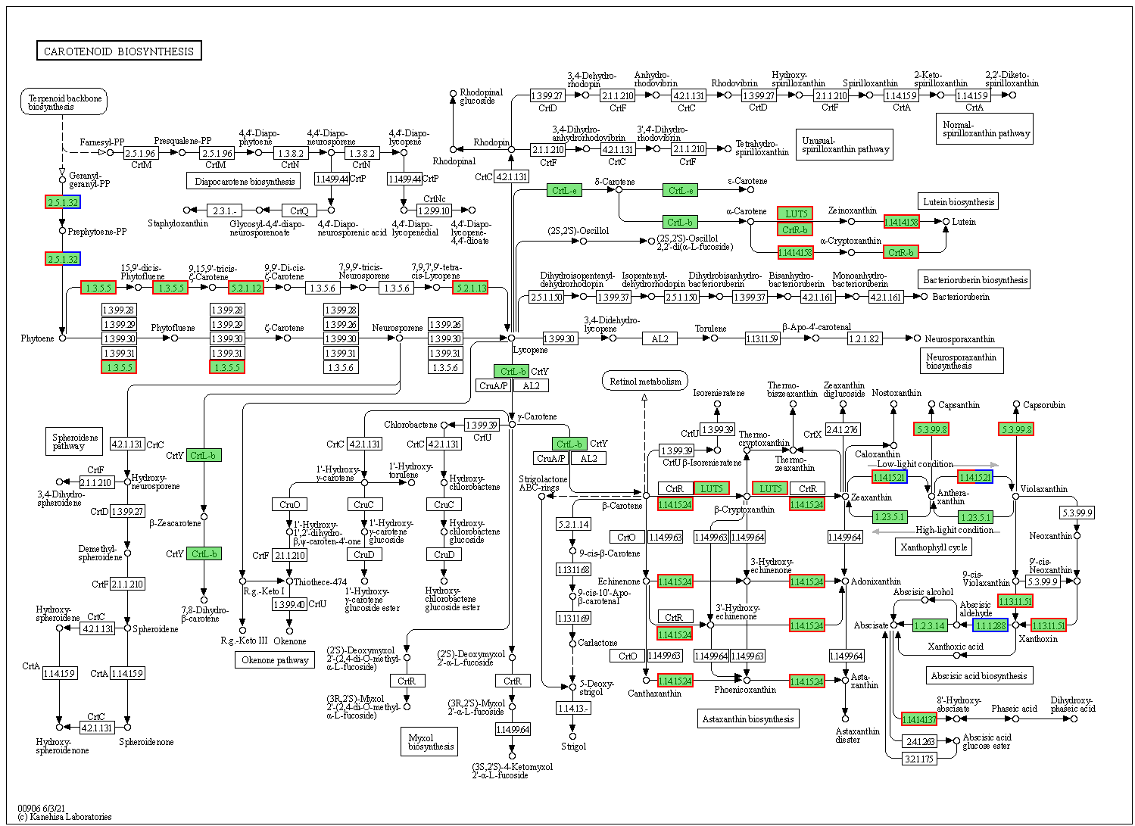


**Figure. S7**


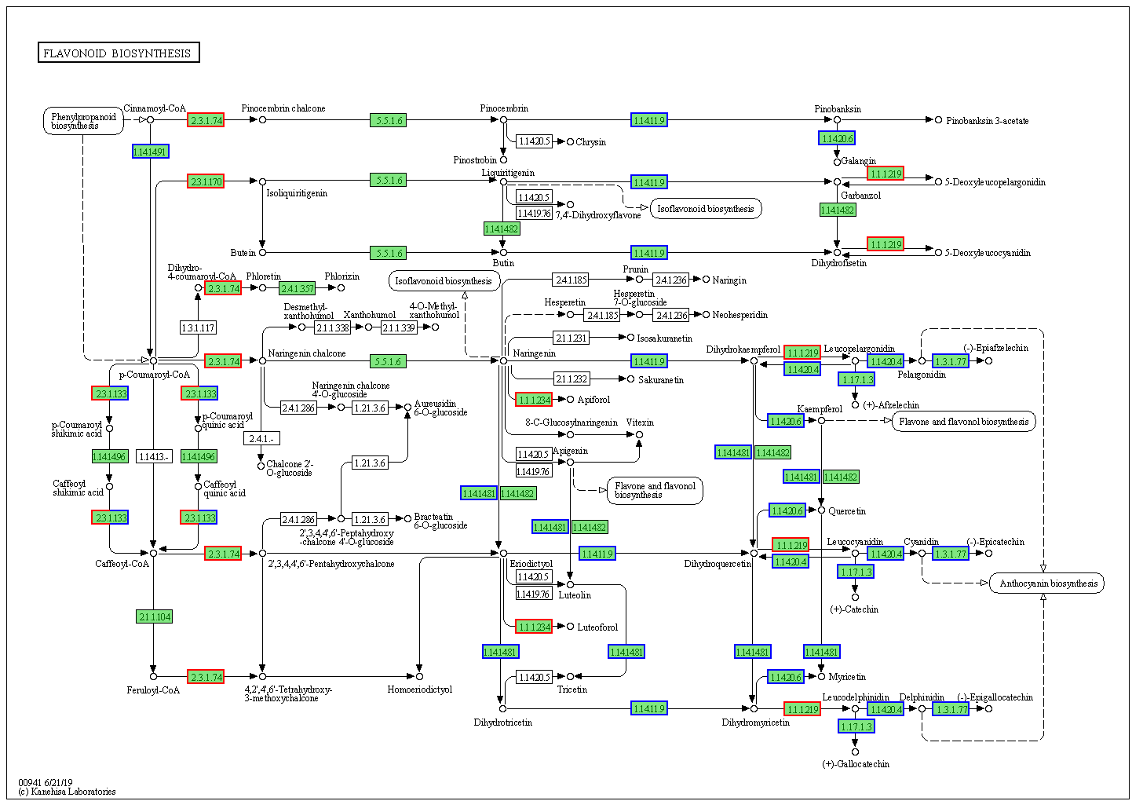


**Figure. S8**


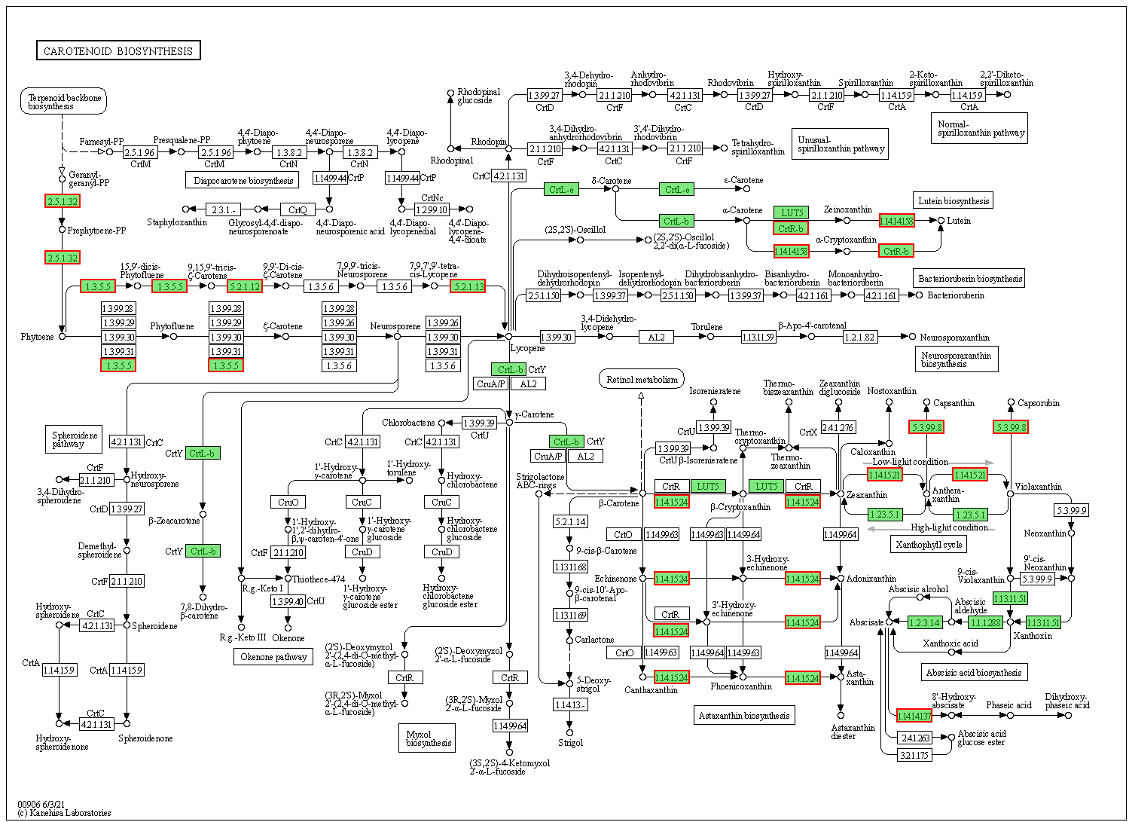


**Figure. S9**


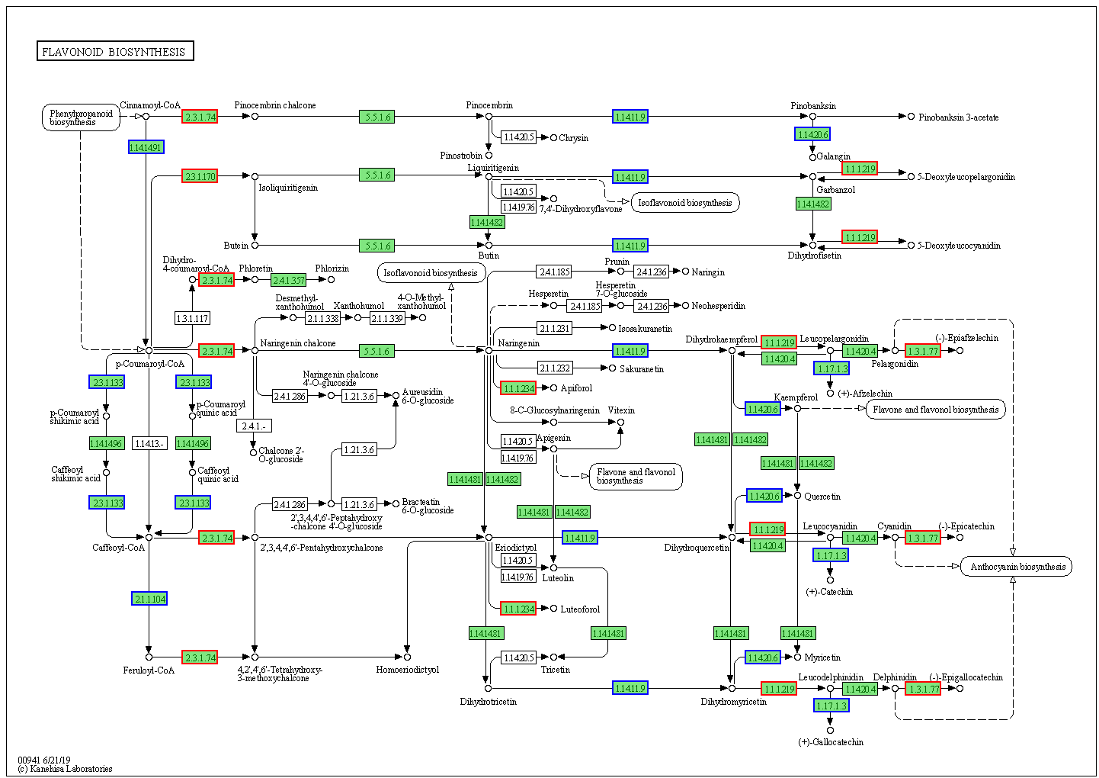


**Figure. S10**


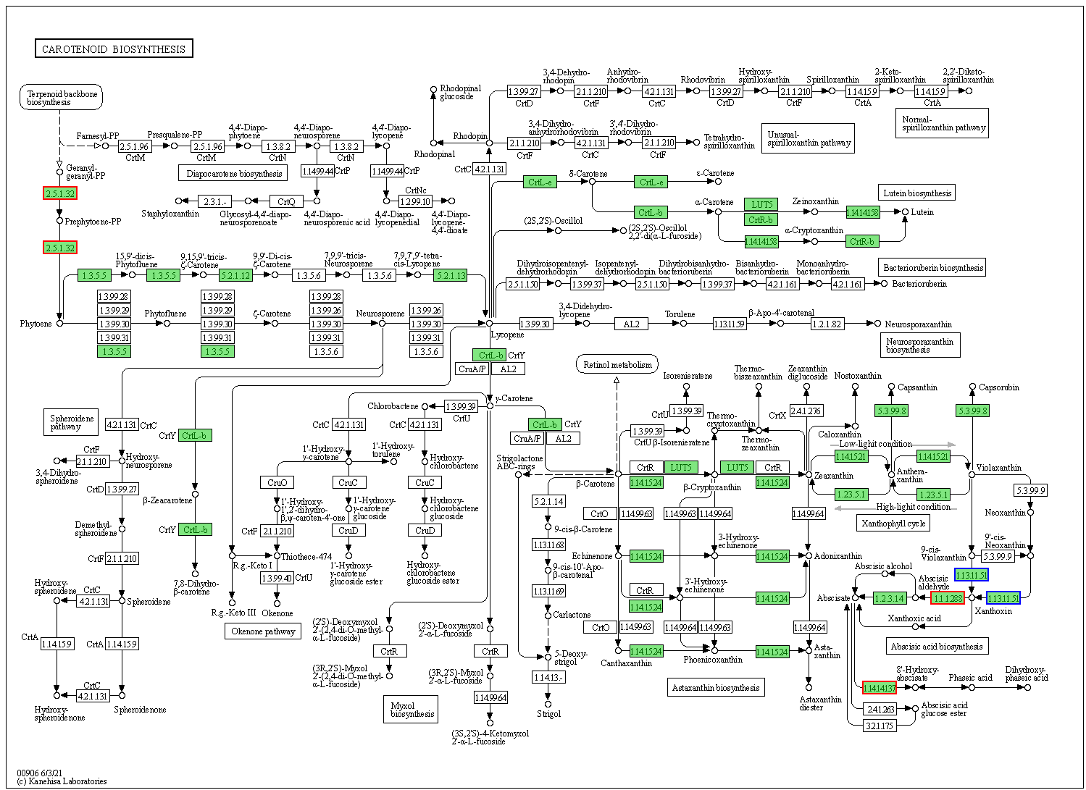


**Figure. S**
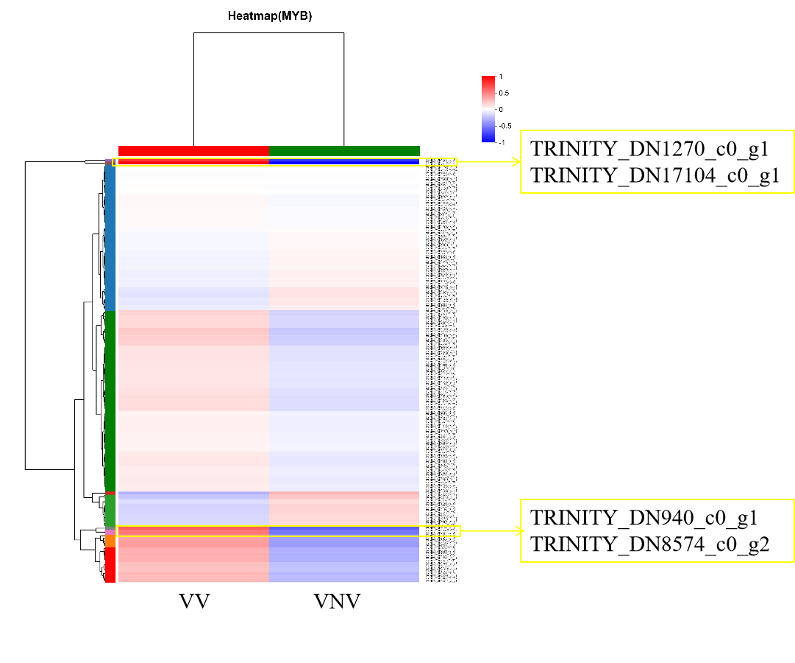
**11**


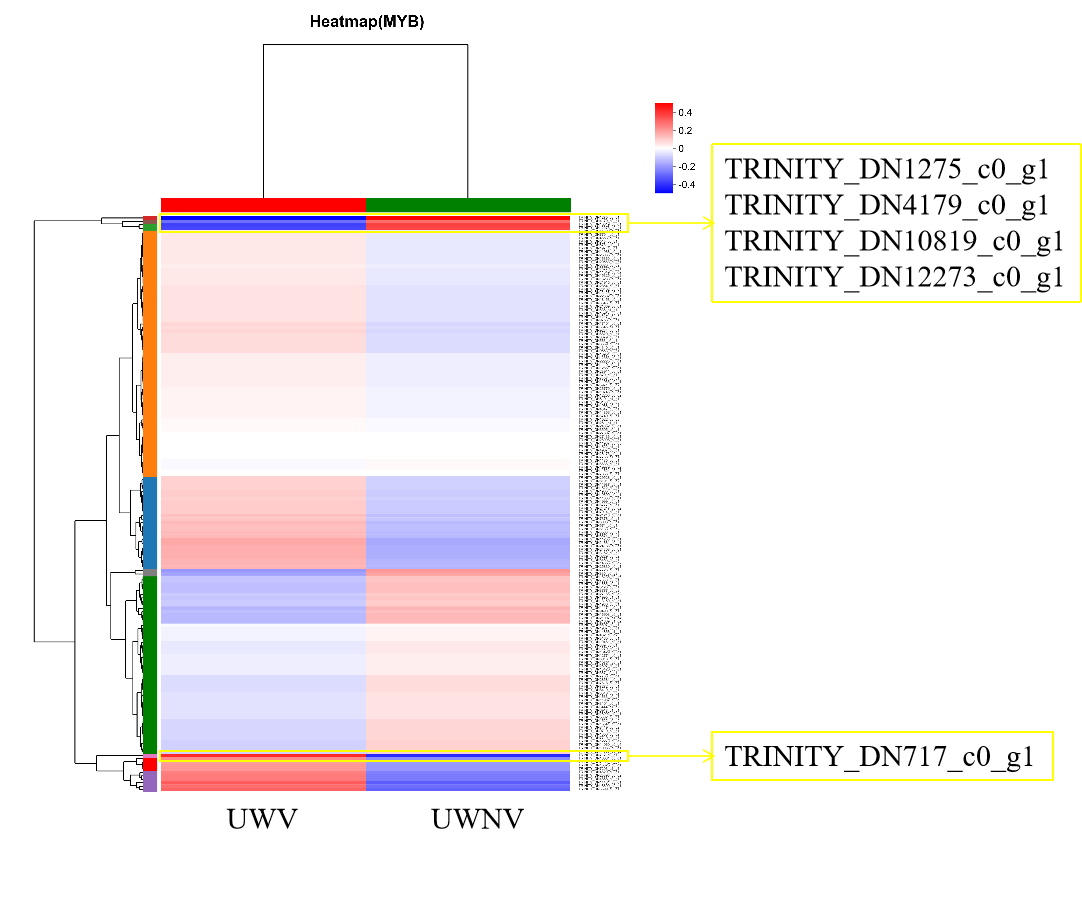
**Figure. S12**

**Figure. S13**


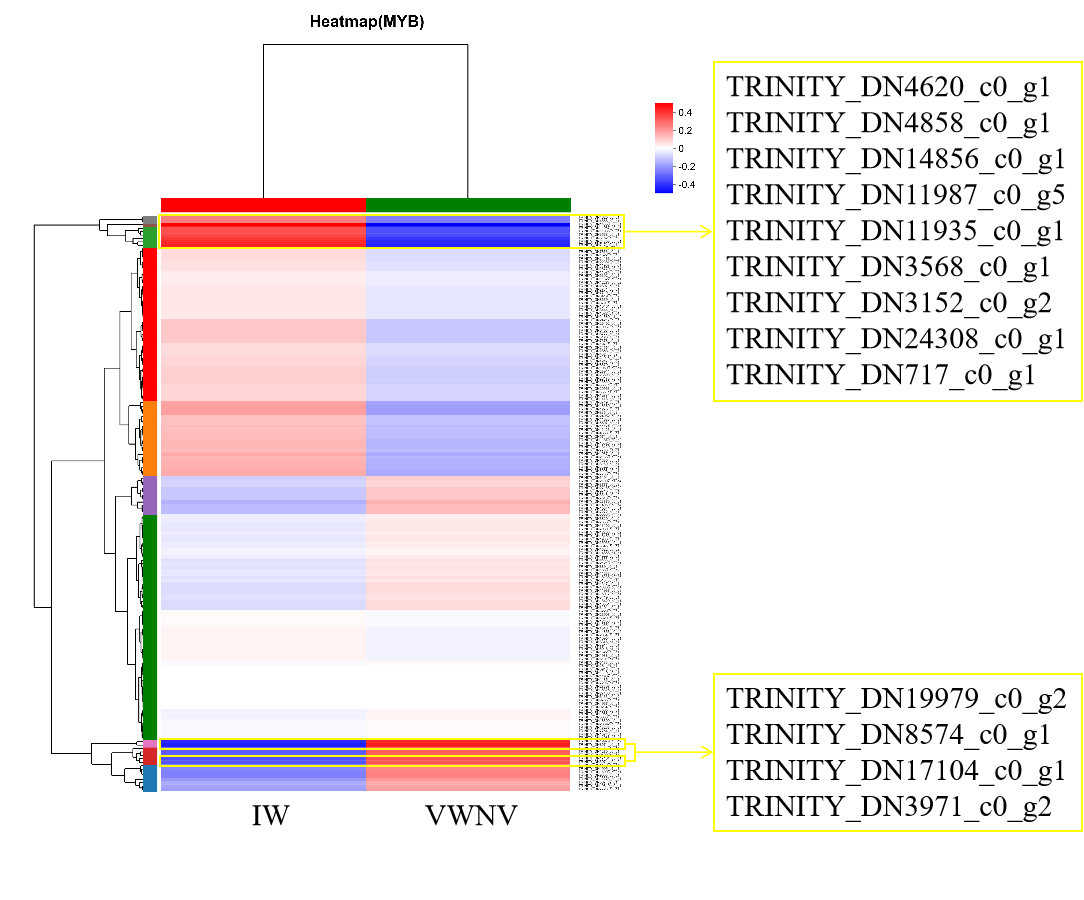


**Figure. S14**


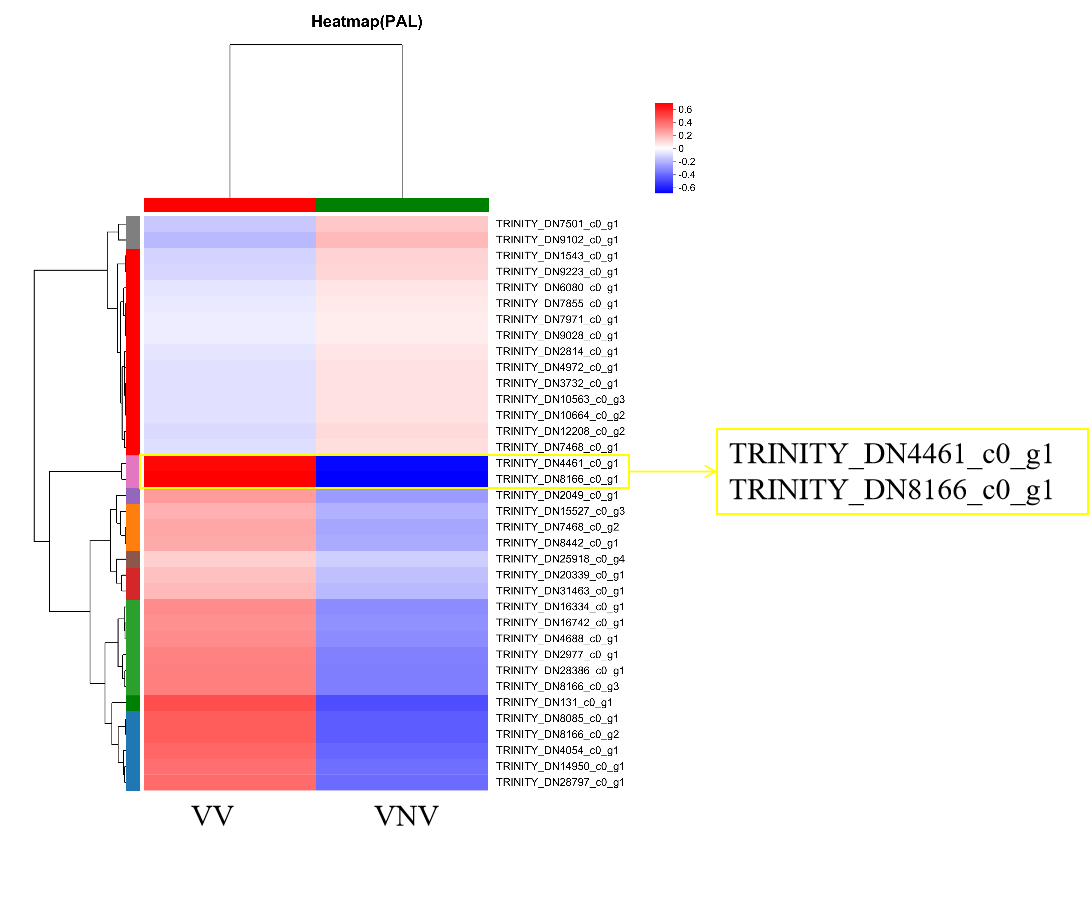


**Figure. S15**


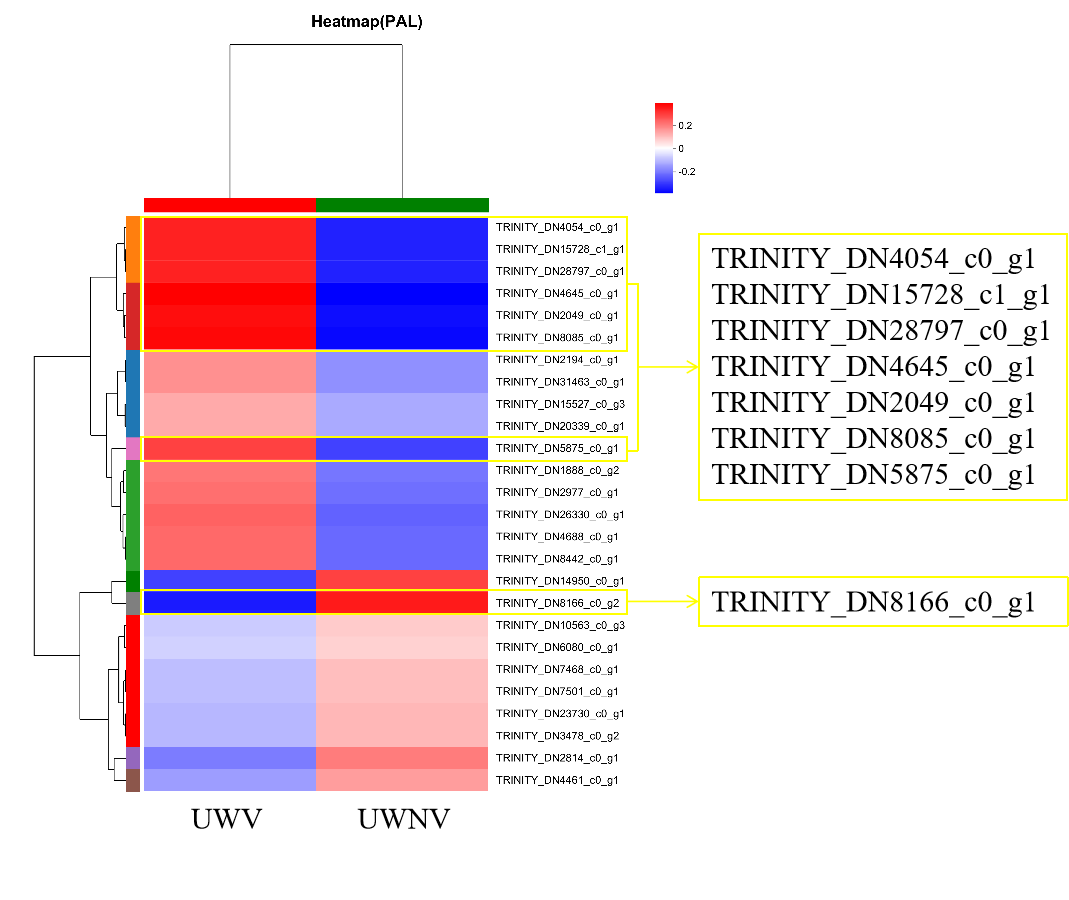


**Figure. S16**


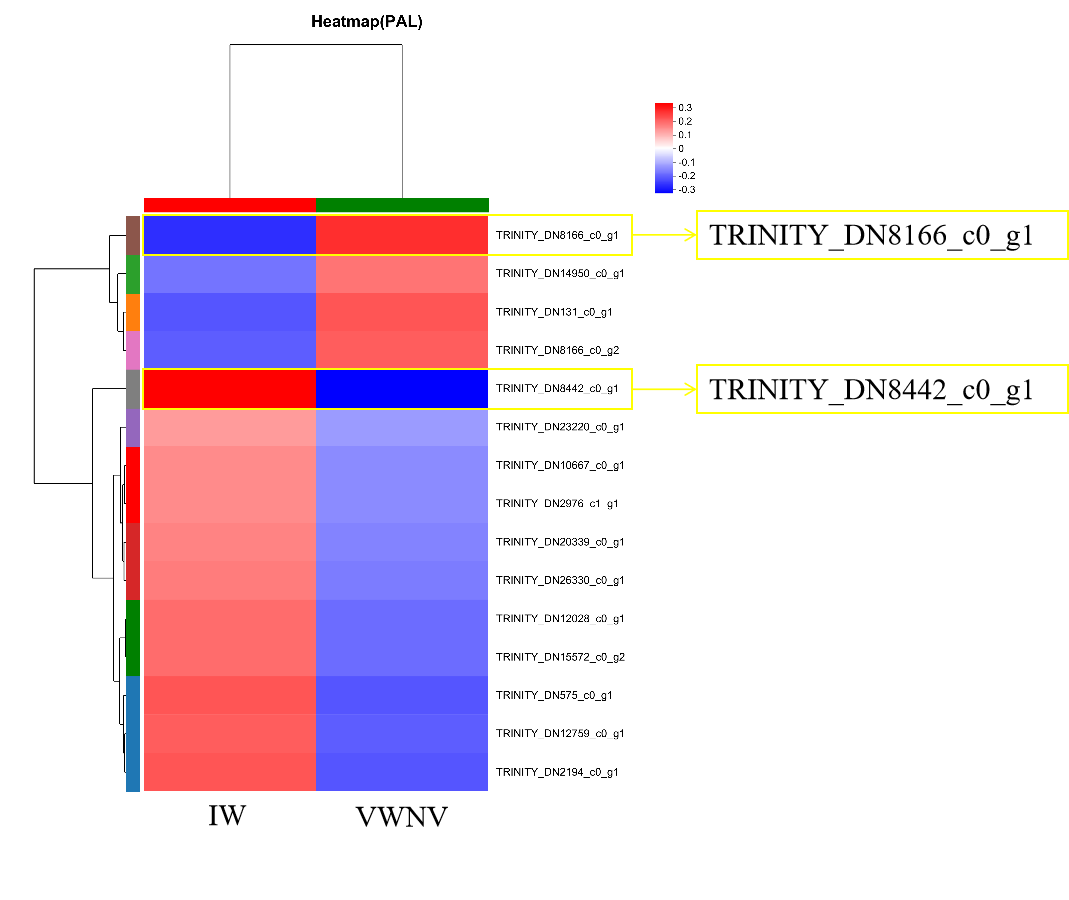


**Supplementary References**

1. Kanehisa, M., Furumichi, M., Sato, Y., Matsuura, Y. and Ishiguro-Watanabe, M.; KEGG: biological systems database as a model of the real world. Nucleic Acids Res. 53, D672-D677 (2025).
2. Kanehisa, M; Toward understanding the origin and evolution of cellular organisms. Protein Sci. 28, 1947-1951 (2019).
3. Kanehisa, M. and Goto, S.; KEGG: Kyoto Encyclopedia of Genes and Genomes. Nucleic Acids Res. 28, 27-30 (2000).

1. [↑](#footnote-ref-0)
